# Harmonizing Rigidity and Flexibility: Embedding COF Quantum Dots Into Extracellular Matrix Gel as Carbon Monoxide Depot for Acoustically Triggered Time‐Programmable Anti‐Infective Therapy

**DOI:** 10.1002/advs.76681

**Published:** 2026-07-20

**Authors:** Baohong Sun, Fang Han, Chunxiao Zhu, Zhiyuan Yang, Haoru Wang, Jinpei Mei, Jie Chen, Jingwen Zhu, Zihan Bo, Taju Wu, Xiaogang Zhou, Tao Ma, Yutian Su, Youhui Lin

**Affiliations:** ^1^ Interdisciplinary Eye Research Institute (EYE‐X Institute) Anhui Engineering Technology Research Center of Biochemical Pharmaceutical School of Pharmacy Bengbu Medical University Bengbu People's Republic of China; ^2^ Department of Physics Research Institute for Biomimetics and Soft Matter Fujian Provincial Key Laboratory for Soft Functional Materials Research Xiamen University Xiamen People's Republic of China; ^3^ State Key Laboratory of Respiratory Disease National Clinical Research Center for Respiratory Disease Guangzhou Institute of Respiratory Health National Center for Respiratory Medicine The First Affiliated Hospital of Guangzhou Medical University Guangzhou People's Republic of China; ^4^ School of Life Science Bengbu Medical University Bengbu People's Republic of China; ^5^ Anhui Key Laboratory of Infection and Immunity School of Basic Medicine Bengbu Medical University Bengbu People's Republic of China

**Keywords:** anti‐inflammatory therapy, carbon monoxide, covalent organic frameworks, extracellular matrix, sonodynamic treatment

## Abstract

Multidrug‐resistant (MDR) bacterial infections persist as a critical threat due to the dual challenge of pathogen burden and challenging post‐infection tissue repair. Herein, this work presents an acoustically triggered carbon monoxide (CO) depot based on rigid‐flexible integrated polymer networks that are engineered for high‐capacity storage and sustained release of CO for infection‐to‐regeneration. This depot is architected by embedding CO precursor‐loaded metallized covalent organic framework quantum dots (COFQDs) into a decellularized extracellular matrix (ECM) gel network derived from small intestinal submucosa (SIS). The rigid COFQDs with abundant Mn sites enable substantial CO storage after ultrasound, while the flexible ECM acts as a gate that slowly enzymatically degrades for CO release up to 10 days. Upon ultrasound activation, the depot eradicates pathogens via sonodynamic therapy, while the liberated CO suppresses the NF‑κB/MAPK/IRF3 axes to remodel inflammation and synergizes with SIS‑derived growth factors to initiate tissue regeneration. In a murine methicillin‐resistant *Staphylococcus aureus* (MRSA)‐infected wound model, this depot fulfilled superior scarless healing compared to conventional treatments. This rigid‐flexible synergistic design establishes a compelling paradigm for temporally regulated anti‐infective therapy, offering a promising strategy for managing MDR diseases.

## Graphical abstract

A rigid‑flexible integrated CO depot is engineered by embedding rigid COFQDs into a flexible ECM gel network. The rigid framework enables high‑capacity CO storage and sonodynamic bacterial killing, while the flexible ECM gel controls sustained CO release, collectively eradicating multidrug‑resistant infection and promoting scarless healing.

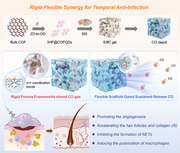

## 1 Introduction

Multidrug‐resistant (MDR) bacteria are a leading cause of severe infections, with over 25% of patients in intensive care units affected, leading to high mortality [1, 2]. These pathogens employ complex, multi‐mechanistic defenses, including biofilm formation, that render single‐antibiotic therapies ineffective [3]. Crucially, antibiotics fail to address the severe tissue inflammation triggered by MDR infections [4]. Bacterial debris/components, such as lipopolysaccharide, are released into the bloodstream, initiating a destructive inflammatory cascade [5]. This prompts massive immune cell recruitment and pro‐inflammatory factor release, damaging vascular and epithelial barriers [6]. Such excessive inflammation can be fatal, causing cytokine storms, multi‐organ failure, and in cases of severe pneumonia or sepsis, respiratory or circulatory collapse [7]. The standard clinical response involves glucocorticoids to suppress inflammation broadly [8]. Nevertheless, this non‐specific immunosuppression fails to restore immune balance or eliminate the inflammatory source [9, 10]. Therefore, there is an urgent need for novel strategies that can not only eradicate multidrug‐resistant bacteria in the early stages of infection but also, more importantly, precisely modulate the immune response in later stages to resolve inflammation.

In recent years, emerging biologic agents (e.g., anti‐TNF‐α monoclonal antibodies, anti‐IL‐1β monoclonal antibodies) and immune agonist delivery systems have brought hope for anti‐inflammatory therapy. However, these anti‐inflammatory immunotherapies only systemically block specific inflammatory factors and fail to intelligently regulate the inflammatory microenvironment to promote its timely resolution. Carbon monoxide (CO) possesses well‐established immunomodulatory effects mediated through the heme oxygenase‐1 (HO‐1) pathway, which is associated with the cell's adaptive response to stress stimuli and injury [11]. In preclinical models, exogenous CO administration is beneficial for treating a variety of diseases, including cardiovascular diseases, sepsis and shock, acute lung injury, and bacterial infections [12]. A substantial body of literature indicates that during stress and inflammation, the delivery of exogenous CO can trigger a series of cytoprotective mechanisms, activating necessary endogenous defenses and anti‐inflammatory functions [13]. However, much work has focused on the untargeted systemic delivery of CO‐releasing molecules, such as transition metal carbonyl complexes [14]. The rapid release and escape of CO gas molecules prevent them from reaching the suitable concentration range for effective action, which reduces therapeutic efficacy and increases systemic toxicity [15]. Furthermore, excessive endogenous CO production within cells may lead to mitochondrial damage, exacerbating the inflammatory response [16]. This leads us to consider that achieving satisfactory clinical outcomes by merely combating oxidative stress through traditional CO generation methods is challenging, highlighting the critical importance of CO collection and controlling the release from depots.

Covalent Organic Frameworks (COFs), as crystalline porous materials, possess tunable structural advantages in the field of gas storage and separation [17]. Their modular design, structural robustness, and controllable pore environment are regarded as ideal reservoirs for CO storage and release [18]. For example, Zhang's team synthesized ICOFs with ultra‐high precision pore tunability via dynamic boronate ester chemistry, enabling precise recognition of different gas molecules [19]. Currently, COFs are primarily used for capturing exogenous gas molecules followed by desorption and recovery; however, this strong affinity‐based adsorption strategy often leads to desorption difficulties [20]. COFs reliant on stimulus‐responsive modules (such as light and ultrasonic) can enable the in‐situ generation and storage of CO gases within their pores, allowing for relatively facile desorption [21, 22]. Furthermore, the large size of COFs severely limits their biomedical application, including challenges such as inefficient cellular uptake and difficulties in metabolic degradation and clearance [21]. Therefore, exfoliating COFs into 0D materials can enhance their biocompatibility and increase the proportion of exposed surface atoms, thereby enhancing gas storage capacity.

However, ultrasmall‐sized COFs are prone to nonspecific aggregation in physiological environments, leading to reduced specific surface area and masked active sites, which severely hinders their responsiveness to external stimuli and the controllability of CO release [23]. The macromolecular network of natural extracellular matrix (ECM), particularly decellularized ECM, can uniformly disperse and immobilize COF quantum dots within its 3D network through physical embedding and chemical interactions [24, 25]. This forms a native, biocompatible, rigid‐flexible COFs array, in which the flexible shell prevents aggregation and enables full functionality. Furthermore, endogenous anti‐inflammatory factors contained in ECM, such as transforming growth factor (TGF‐β) and vascular endothelial growth factor (VEGF), can synergize with the anti‐inflammatory effects of CO, more efficiently regulating immune cells and alleviating excessive inflammation [26].

**SCHEME 1:**
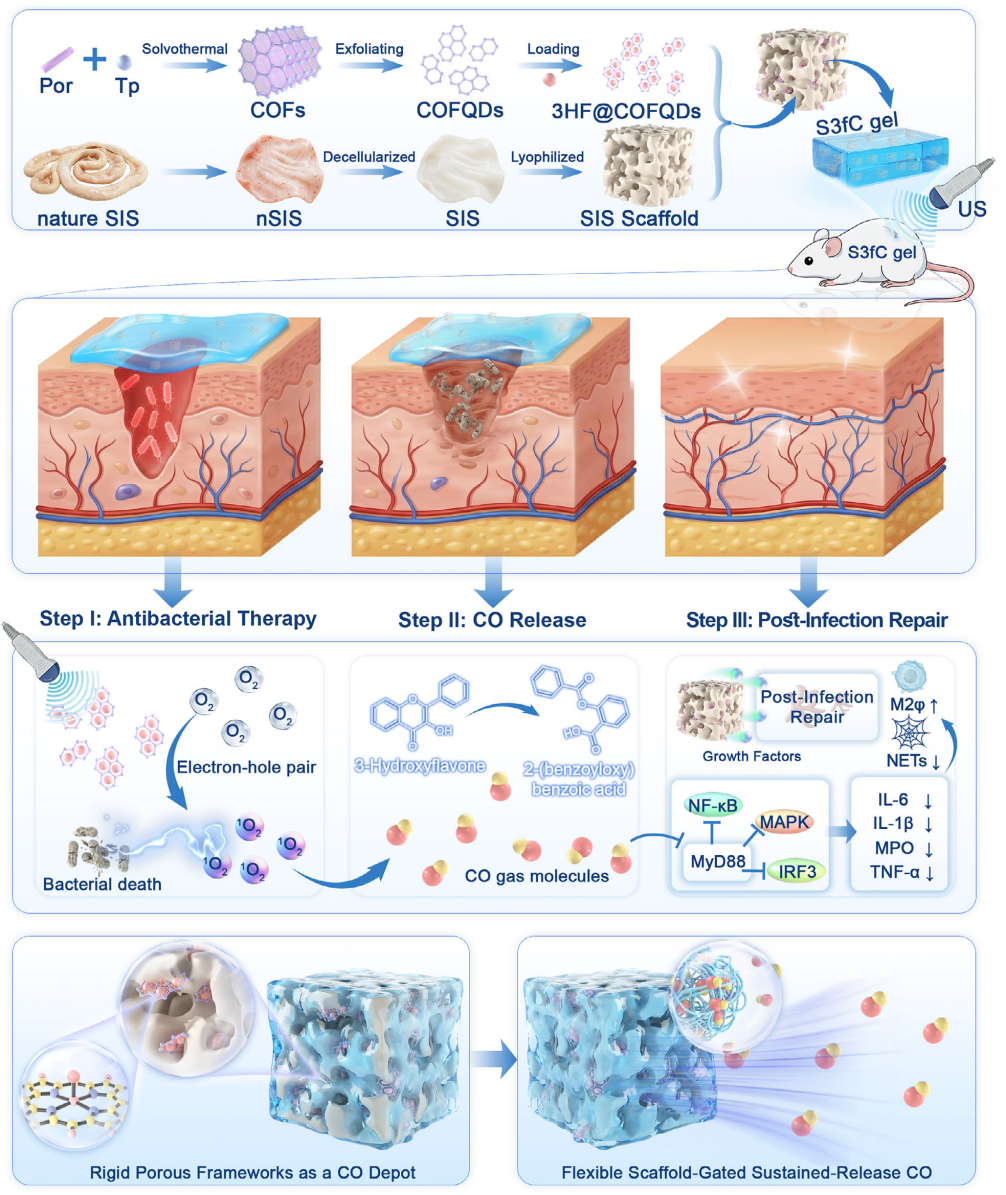
Schematic of the CO depot (S3fC gel) fabrication and its integrated repair strategy against infections, combining MDR eradication, cascaded CO release, and SIS gel for anti‐inflammatory activity and high‐quality infected tissue regeneration.

In this study, an ultrasound‐triggered CO depot was engineered via a rigid‐flexible integrated strategy, wherein rigid COF quantum dots (COFQDs) cores were sheathed within a flexible ECM macromolecular network, enabling temporally regulated anti‐infective therapy (Scheme 1). To implement this design, a porphyrin manganese (Mn)‐immobilized sonosensitive COF linked via boronate ester bonds was first synthesized. The COF was then exfoliated into quantum dots through a self‑hydrolysis strategy, while the 3‑hydroxyflavone was uniformly dispersed into the pores, yielding 3HF@COFQDs. Subsequently, 3HF@COFQDs were embedded into a decellularized porcine small intestinal submucosa (SIS) ECM to form the in situ‑assembled CO depot (designated S3fC gel). Upon ultrasound (US) activation, the COFQDs generate reactive oxygen species (ROS) within their pores, which in turn trigger the production of CO from 3HF. The released CO signaling molecules strongly inhibit the pro‐inflammatory pathway and cooperate with cytokines in the ECM to repair tissues. Ultimately, the S3fC gel not only clears multidrug‐resistant pathogens in the early stage via sonodynamic therapy (SDT), but also remodels the inflammatory microenvironment in the later stage to achieve high‐quality post‐infection repair. During this process, the COF‐ECM double‐crosslinked network functions as an integrated storage‐release system: the rigid pores provide precise storage for CO gas with Mn acting as the adsorption sites, while the flexible polymer network serves as a smart gate, controlling the release of CO through its own degradation. Together, these results demonstrate that the S3fC gel represents a promising therapeutic strategy for treating MDR‑induced infectious diseases, with substantial translational potential.

## 2 Results and Discussion

### 2.1 Synthesis and Characterization of 3HF@COFQDs

**FIGURE 1:**
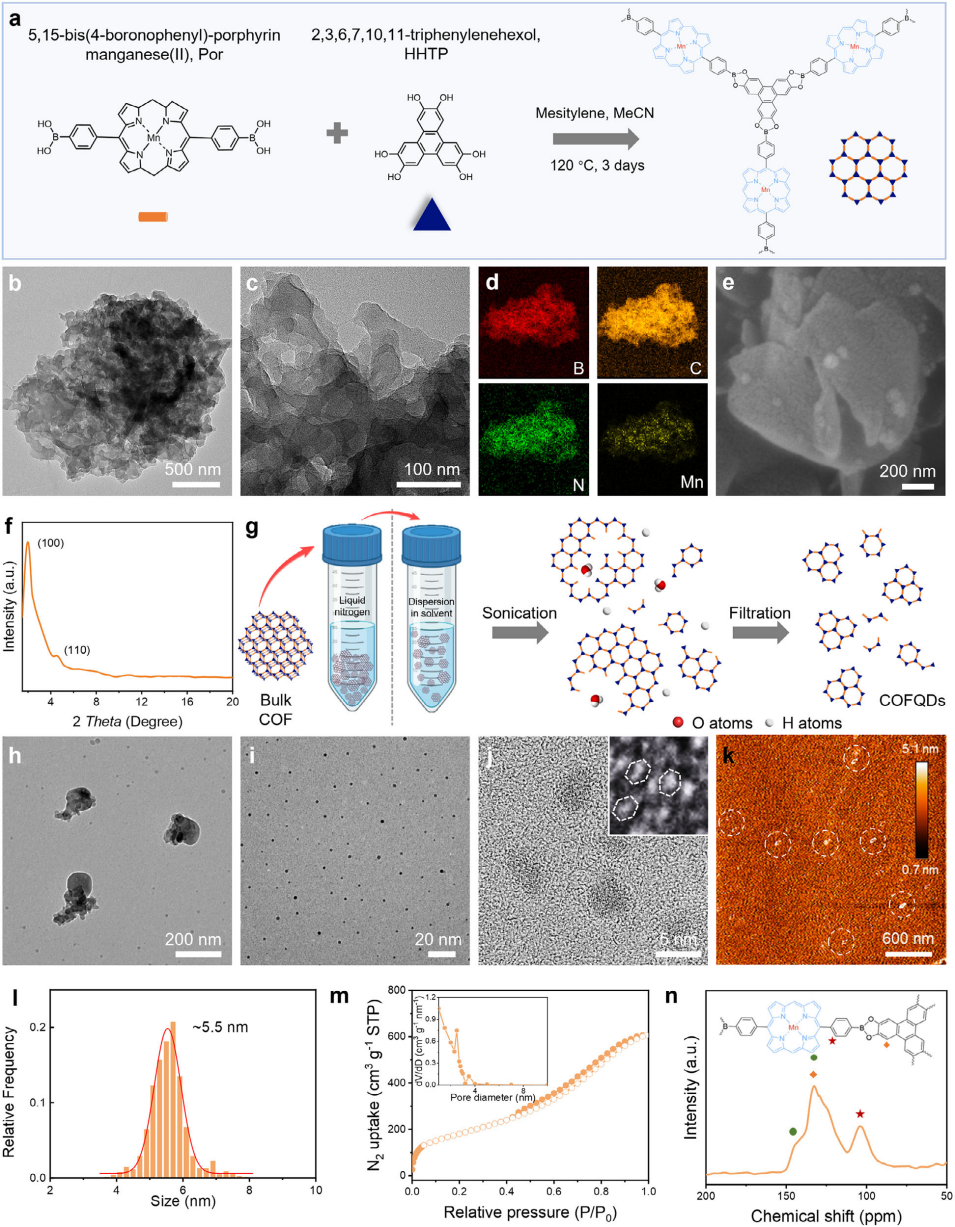
Design and structural characterization of the ultrasmall COFQDs and 3HF@COFQDs. (a) Schematic illustration of the synthetic procedure and topological structure of Mn‐doped COFs. (b, c) HRTEM images of COFs. (d) TEM‐EDX mapping images of B, C, N, and Mn elements in selected areas of COFs. (e) SEM image of COFs. (f) XRD pattern of COFs. (g) Schematic illustration of the self‐hydrolysis synthesis of COFQDs. (h) HRTEM image of transitional product during synthesis of COFQDs. (i, j) HRTEM images of COFQDs. Insert: hexagonal channels of COFQDs. (k) AFM image of COFQDs. (l) DLS analysis of COFQDs. (m) BET adsorption isotherm and pore size distribution of COFQDs. (n) ^13^C CPMAS spectrum of COFQDs.

Our investigation into the synthesis of COFs began with an attempt to synthesize a boronate ester‐linked COF via condensation polymerization using 5,15‐bis(4‐boronophenyl)‐porphyrin manganese(II), (Por) and 2,3,6,7,10,11‐triphenylenehexol (TP) (Figure 1a). Expectedly, reacting Por with TP in the presence of mesitylene and acetonitrile yielded COFs (TP‐Por), a boronate ester‐linked product along with water molecules. The representative high‐resolution transmission electron microscopy (HRTEM) images show that the horizontally oriented COFs are approximately a few micrometers and have small‐scale flakes with an open porous network (Figure 1b,c). Chemical mapping analysis via energy‐dispersive X‐ray (EDX) spectroscopy revealed that the C, B, O, and Mn components are uniformly distributed within the COF sheets (Figure 1d). The X‐ray absorption near‐edge structure (XANES) and extended X‐ray absorption fine structure (EXAFS) spectra confirmed the existence of the metal center in a Mn–N coordination motif (Figure S1). Scanning electron microscopy (SEM) analyses of the COFs display an agglomeration of lamellar‐like crystallites (Figure 1e). Powder X‐ray diffraction (PXRD) analysis confirmed the high crystallinity of the synthesized COFs. The most intense peak for COFs appears at 2.0° (2θ), and another peak at 4.5° (2θ), accountable for the reflections from the (100) and (110) planes (Figure 1f). The calculated Brunauer–Emmett–Teller (BET) surface areas are found to be 324.7 m^2^/g with a type‐I(b) isotherm for COFs. Evaluation of pore size distribution by the density functional theory (DFT) method results in the distribution peak for COFs at 4.53 nm (Figure S2a).

2D COFs tend to aggregate in physiological environments, compromising their functionality and controllability. Therefore, we exfoliated 2D‐scaled COFs into 0D‐scaled COFQDs via liquid nitrogen cryo‐assisted cryo‐grinding and high‐power ultrasonication (US) in an acidic medium based on a self‐hydrolysis strategy. During this process, the boronate ester bonds in the COFs cleave, forming dimers and other oligomers (Figure 1g). The mechanism involves protonation of the boronate ester bond, which enhances the electrophilicity of the Lewis acidic boron center and facilitates nucleophilic attack by water, leading to hydrolytic cleavage [27, 28]. The kinetic process of hydrolysis is driven by the synergy between the electron‐deficient nature of trivalent boron and the acid‐induced protonation of oxygen, enabling rapid and selective bond cleavage [29]. Next, we used TEM to monitor the progression of COFQD formation. It was observed that the several‐micrometer‐sized COFs were exfoliated down to the nanoscale after US, accompanied by the generation of small‐sized quantum dots (Figure 1h). Upon completion of US, the incompletely degraded COF fragments were filtered to obtain relatively uniform‐sized COFQDs (Figure 1i). HRTEM revealed that the COFQDs exhibited an irregular spherical morphology with distinct edges (Figure 1j). Also, hexagonal grids observed via cryo‐TEM can provide confined spaces for CO‐releasing molecules to undergo chemical reactions. Atomic force microscopy indicated a height of approximately 5.0 nm (Figure 1k), which was consistent with the particle size statistics obtained from HRTEM (Figure 1l). The Tyndall effect serves as a well‐established phenomenon for assessing particle size in colloidal dispersions. The presence of a clear Tyndall effect in the aqueous dispersion of COFQDs further confirmed that the COFs had been exfoliated down to colloidal dimensions (Figure S3). Interestingly, the drastic change in ζ‐potential from +5 mV to −26 mV (Figure S4) strongly supports the conversion from a 2D to a 0D structure. Compared with 2D material, the 0D quantum dots possess a vastly increased specific surface area and a high density of edges [30]. The reduced steric hindrance at these sites allows the hydroxyl groups to undergo deprotonation more readily, contributing to a greater negative charge [31]. As the dimension decreases, the BET specific surface area progressively increases to 684.8 m^2^/g, while the intrinsic porous characteristics of the COF material are preserved (Figure 1m). After immersion in a mildly acidic solution for 7 days, the entire framework undergoes complete degradation, indicating its advantageous biocompatibility over its 2D counterpart (Figure S5).

Fourier transform infrared (FT–IR) spectroscopy analyses displayed signals at ∼1440–1300 cm^−1^ and ∼800–700 cm^−1^ region for the COFQDs, indicating the presence of boron‐carbon bonds (−B−C) and porphyrin ring breathing vibration (−Mn−N) [32]. Distinct signals at ∼3664–3090 cm^−1^ are observed, which are attributed to hydroxy −OH stretching (Figure S6). The high‐resolution X‐ray photoelectron spectroscopy (XPS) of C 1s for COFQDs is deconvoluted to two peaks at binding energies of ∼289.6 and ∼284.8 eV, corresponding to C−B and C−C bonds of the frameworks (Figure S7a). Besides, N 1s high‐resolution spectra of COFQDs exhibit two distinct peaks at binding energies of ∼398.2 and 400.4 eV, which are attributed to the C═N and Mn−N in porphyrin rings, respectively (Figure S7b), whereas for O 1s, only one peak at ∼532.4 eV is observed for oxygen‐boron bonds (Figure S7c) [33]. Further investigation through solid‐state ^13^C cross‐polarization magic‐angle spinning (CPMAS) nuclear magnetic resonance spectroscopy presents the ^13^C signals at ∼143.3 ppm (for porphyrin rings), ∼132.4 ppm (for triphenylene), and ∼103.5 ppm (for benzene rings), which confirms the existence of boronate ester linkage (−C═C−B−O−C═C) bonds (Figure 1n) [34]. These data demonstrate that the network chemical structure of COFs is effectively preserved after exfoliation into quantum dots.

Subsequently, a nanodrug designated as 3HF@COFQDs was constructed by encapsulating the CO‐releasing precursor into the confined cavities of the COFQDs via host‐guest interactions, achieving controllable CO release. Nitrogen adsorption‐desorption experiments revealed that after the incorporation of 3HF into the COFQDs cavities, the BET specific surface area and cumulative pore distribution decreased significantly to 387.8 m^2^/g and 1.1 nm, respectively (Figure S2b). Furthermore, the shape of the isotherm changed, indicating successful ingress of 3HF into the interior of COFQDs. Similarly, the ζ‐potential of 3HF@COFQDs increased to −17 mV, a change likely owing to the surface adsorption of 3HF molecules compared with the pristine COFQDs (Figure S4). FT‐IR spectroscopy provided direct evidence for the effective conjugation between the CO‐releasing molecule and COFQDs. The absorption bands at ∼3010–2838 cm^−1^ and ∼1504–1326 cm^−1^ were assigned to the C−H and C = O stretching vibrations, respectively, both characteristic of the 3HF molecule (Figure S6). Unexpectedly, the loading capacity of COFQDs for the 3HF precursor, as determined by high‐performance liquid chromatography (HPLC), reached a remarkable 67.2 wt%. Based on these results, we successfully employed a self‐hydrolysis strategy to construct the ultrasmall nanodrug 3HF@COFQDs, paving the way for the subsequent establishment of a CO depot.

### 2.2 Sonodynamic Performance and CO Cascade Release of 3HF@COFQDs

**FIGURE 2:**
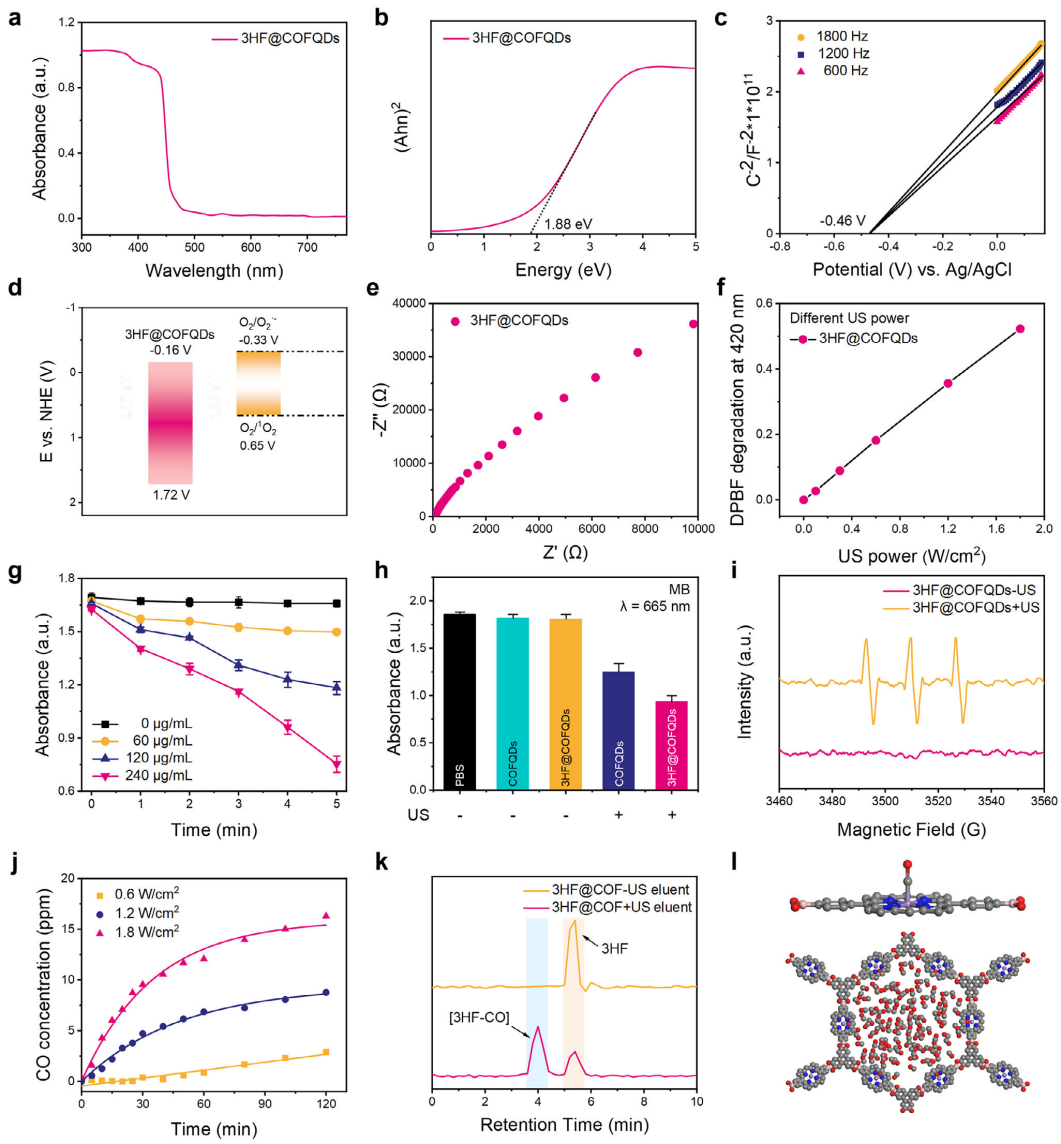
Characterization of the SDT and CO‐release properties of 3HF@COFQDs. (a) UV‐vis DRS spectrum of 3HF@COFQDs. (b) Tauc plot of 3HF@COFQDs. (c) Mott‐Schottky plots for 3HF@COFQDs. (d) The intrinsic energy bands for 3HF@COFQDs. (e) The impedance values of 3HF@COFQDs. (f) DPBF assay for assessing the SDT activity of 3HF@COFQDs. (g) Analysis of ROS generation efficiency of 3HF@COFQDs at various concentrations using the DPBF probe. n = 3. (h) Analysis of the degradation efficiency of COFQDs and 3HF@COFQDs under different US conditions via the MB probe assay. n = 3. (i) ESR spectrum of 3HF@COFQDs under different US conditions. (j) CO release kinetics from 3HF@COFQDs under varying US power. (k) HPLC analysis of 3HF@COFQDs in the eluent, pre‐ and post‐US irradiation. (l) Model illustrating CO distribution in 3HF@COFQDs pores and its binding to Mn sites.

Given the key role of US‐responsive COFs in building in situ CO depots, the sonodynamic performance of 3HF@COFQDs was tested. UV‐visible (UV‐Vis) diffuse reflectance spectroscopy revealed that 3HF@COFQDs exhibit distinct absorption peaks in the visible light range from 300 to 500 nm (Figure 2a), indicating excellent visible light absorption efficiency. The Tauc plot was used to determine the band gap (Eg) of 3HF@COFQDs as 1.88 eV (Figure 2b). According to the Mott‐Schottky plot (Figure 2c), the flat‐band potential of 3HF@COFQDs relative to the Ag/AgCl electrode is ‐0.46 V. Using the formula E_VB_ = E_CB_ + E_g_, the valence band potential (EVB) of 3HF@COFQDs was estimated to be 1.72 V [35]. After normalization against the standard hydrogen electrode (NHE, pH 6.8), the calculated conduction band potential (ECB) was ‐0.16 V (Figure 2d). The redox potential of O_2_/singlet oxygen (^1^O_2_) is −0.33 V. The conduction band edge of 3HF@COFQDs possesses a more positive redox potential than that of O_2_/^1^O_2_, which favors the generation of ^1^O_2_ under ultrasonic irradiation. US‐induced charge separation and transfer are crucial for the performance of sonosensitizers. Electrochemical impedance spectroscopy (EIS) showed a lower charge transfer resistance for 3HF@COFQDs, suggesting superior charge separation capability (Figure 2e). The efficiency of electron‐hole pair separation is vital for the activity of sonosensitizers. Further investigation by sonoelectrochemistry demonstrated a rapid increase in current for 3HF@COFQDs upon US irradiation, highlighting the strong electron transfer capacity of the porphyrin‐based 3HF@COFQDs (Figure S8).

Having confirmed the theoretically excellent sonosensitizing property of 3HF@COFQDs, we employed various radical probes to clarify their reactive oxygen species (ROS) generation capacity under US irradiation. First, using 1,3‐diphenylisobenzofuran (DPBF) as an indicator, the sonodynamic effect of 3HF@COFQDs was investigated. We found that 3HF@COFQDs exhibited satisfactory DPBF degradation efficiency under ultrasonic conditions (Figure 2f). The degradation efficiency of DPBF by 3HF@COFQDs was subsequently measured at different concentrations, showing a clear concentration dependence (Figure 2g). At a concentration of 240 µg/mL, 3HF@COFQDs achieved near‐complete degradation of DPBF within 5 min under US irradiation. We next employed methylene blue (MB) as a probe to assess potential differences in the sonocatalytic performance between COFQDs and 3HF@COFQDs (Figure 2h). The results showed that neither material produced ROS nor degraded MB in the absence of US. However, upon ultrasonic irradiation, both COFQDs and 3HF@COFQDs facilitated effective degradation, demonstrating that the incorporation of 3HF does not compromise the intrinsic sonodynamic performance of COFQDs. Next, electron spin resonance (ESR) spectroscopy was used to analyze the radical species (Figure 2i). A characteristic 1:1:1 triplet signal was observed for the 3HF@COFQDs+US group, while no such signal was detected for the 3HF@COFQDs‐US group, indicating that the primary radical species generated under ultrasonic stimulation is ^1^O_2_. In summary, the high electron‐hole separation capability of 3HF@COFQDs makes it a crucial material for subsequent CO cascade‐release experiments, based on its excellent sonodynamic effect.

To gain deeper insight into the cascade reaction of ROS‐triggered CO generation, we investigated the CO concentration from 3HF@COFQDs under different US power levels. Under US at powers of 0.6, 1.2, and 1.8 W/cm^2^ for 120 min, the maximum CO concentrations reached 2.9, 8.8, and 16.4 ppm, respectively, exhibiting both time‐ and power‐dependence (Figure 2j). We found that the CO release at 1.2 and 1.8 W/cm^2^ followed a quasi‐first‐order kinetic model. Subsequently, to determine whether the structure of 3HF changed after CO generation, 3HF@COFQDs were immediately eluted after sonication and analyzed using an HPLC system (Figure 2k). As expected, without sonication, the eluent showed a distinct chromatographic peak at a retention time of 5.4 min. After sonication, this signal significantly weakened, and a new chromatographic peak appeared at a retention time of 4.0 min, which we speculate may correspond to the [3HF−CO] product formed after CO‐releasing molecules generated CO. High‐resolution mass spectrometry analysis of the two eluents revealed that the 3HF@COFQDs‑US group produced an [M+H]^+^ signal at m/z 239.0689, consistent with the theoretical [M+H]^+^ value of 239.24 (Figure S9). In contrast, the 3HF@COFQDs+US group displayed not only the signal at m/z 239 but also a new mass spectrometry peak at m/z 243.3863. Based on the mass spectrometry results, we proposed a possible reaction mechanism: Upon ROS‐mediated oxidation, 3HF undergoes oxidative ring cleavage and decarbonylation, releasing CO and 2‐(benzoyloxy)benzoic acid (theoretical molecular weight: 242.23). Similarly, gas chromatography (GC) confirmed that the gas produced was CO (Figure S10). This is consistent with previous reports showing that 3HF and its derivatives release CO upon ROS stimulation [36, 37, 38, 39, 40, 41]. To directly probe the proposed CO storage mechanism, we performed in situ diffuse reflectance infrared Fourier transform spectroscopy (in situ DRIFTS) on COFQDs after CO loading. The DRIFTS spectra showed a distinct peak at ∼2050 cm^−1^ (Figure S11), characteristic of terminal Mn‑CO stretching. Its position is red‑shifted by nearly 100 cm^−1^ from free CO (2143 cm^−1^), indicating strong π back‑donation from Mn to CO, which is the defining feature of a σ‑π coordination bond [42]. This provides direct spectroscopic evidence for the formation of Mn‑CO σ‑π bonds, supporting the reversible CO storage at Mn sites. Consequently, we confirm that 3HF@COFQDs can function as a novel platform for the cascaded generation of CO upon ultrasound stimulation. The reason behind this is that the Mn elements possess incompletely filled d‐orbitals, enabling the formation of unique σ‐π coordinate bonds with CO molecules (Figure 2l) [43]. When CO molecules diffuse into the rigid pores of the COF and encounter the Mn sites, they are gently chemisorbed and immobilized via these σ‐π bonds, thereby preventing free diffusion and loss [44]. Furthermore, the rigid porous structure of the COFQDs serves as a gas storage medium for holding an ample supply of CO.

### 2.3 Antimicrobial and Antibiofilm Activity of 3HF@COFQDs

**FIGURE 3:**
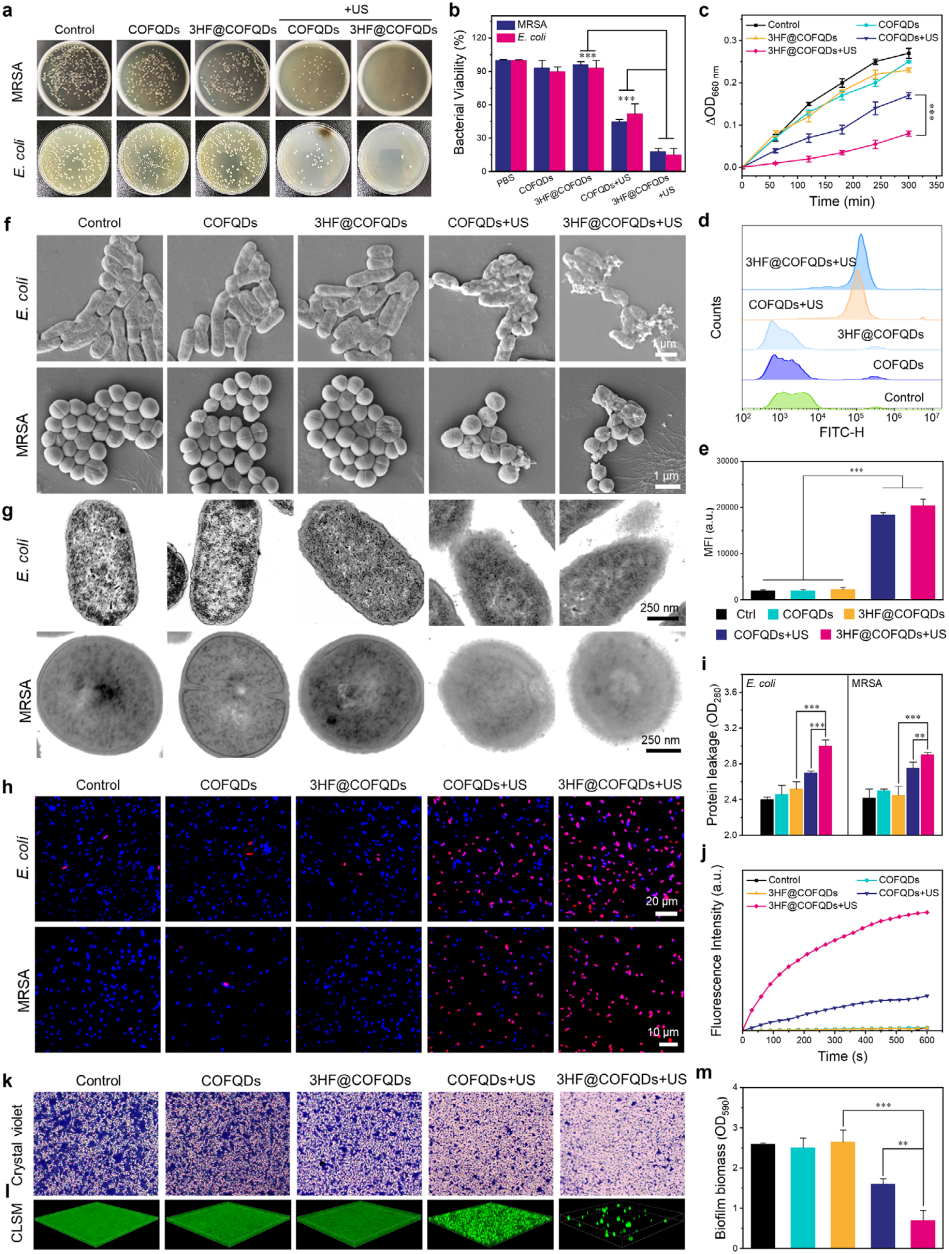
Evaluation of the antimicrobial and anti‐biofilm efficacy of 3HF@COFQDs. (a) Bacterial counting of *E. coli* and MRSA treated by PBS, COFQDs (with or without US), 3HF@COFQDs (with or without US). (b) Assessment of bacterial viability following different treatments. n = 5. (c) Bacterial growth curve changes over a 300‐min period following different treatments. n = 5. (d, e) Flow cytometry analysis and quantification of bacterial endogenous ROS levels. n = 3. (f, g) SEM and TEM images of *E. coli* and MRSA following different treatments. (h) Bacterial permeability dye staining of *E. coli* and MRSA, with blue indicating bacterial DNA/nucleic acids and red indicating membrane rupture. (i) Protein leakage assay of supernatants from both bacterial cultures after treatment with different materials. n = 5. (j) Kinetic analysis of MRSA membrane damage using a fluorescent probe. (k) Representative images of crystal violet staining assessing MRSA biofilm formation under various treatments. (l) Representative images of 3D reconstructions of biofilms stained with SYTO 9 after different treatments. (m) Quantification of biomass in MRSA biofilms after different treatments. n = 3. Data are expressed as mean ± SD. Statistical significance was determined using a two‐sided *t*‐test, * denotes *p* < 0.05, ** denotes *p* < 0.01, *** denotes *p* < 0.001.

In the early stages of infection, timely clearance of pathogenic bacteria to prevent progression into an active inflammatory phase is crucial for tissue repair. Therefore, we evaluated the sonodynamic therapy (SDT) antibacterial performance of 3HF@COFQDs. Gram‐negative *Escherichia coli* (*E. coli*) and Gram‐positive methicillin‐resistant *Staphylococcus aureus* (MRSA) were selected as model bacterial strains. Using the dilution plate counting method, it was found that at a concentration of 150 µg/mL, neither COFQDs nor 3HF@COFQDs alone exhibited any antibacterial effect, consistent with the control group. In contrast, after treatment with COFQDs+US and 3HF@COFQDs+US, the viabilities of the two bacterial strains were reduced to below 45% and 15%, respectively (Figure 3a,b). Live/dead fluorescent staining (using propidium iodide, PI) of the bacterial suspensions subjected to different treatments showed that the intensity trend of red PI fluorescence was consistent with the bacterial colony‐forming unit (CFU) counts (Figure S12). The 3HF@COFQDs+US group demonstrated the highest antibacterial activity, surpassing that of the COFQDs+US treatment group. The underlying reason for this enhancement may be a synergistic effect between CO and ROS generation. Similarly, previous works reported that culturing bacteria in a CO‐pretreated solution did not alter bacterial viability, whereas combining it with reactive nitrogen species increased antibacterial efficiency several‐fold [45]. Subsequently, bacterial growth curves were monitored at different time points after a 30‐min US treatment. Significant differences in growth inhibition among the groups became apparent at the 60‐min mark. An optical density as low as 0.07 was achieved at 300 min for the 3HF@COFQDs+US group, demonstrating a significant reduction compared to the COFQDs+US group (Figure 3c), which is likely attributable to the sustained release of CO. MRSA bacterial suspensions collected after US treatment were subjected to flow cytometric analysis using 2',7'‐dichlorodihydrofluorescein diacetate (DCFH‐DA) as an ROS probe. The results indicated that both materials generated substantial endogenous ROS under US exposure, with levels several orders of magnitude higher than those in the control group. This suggests that the observed pathogen death likely occurred via oxidative stress (Figure 3d,e).

To elucidate the potential antibacterial mechanism, we examined the morphology of the treated bacteria using SEM. In contrast to the smooth, rod‐shaped or oval untreated cells, both *E. coli* and MRSA treated with the 3HF@COFQDs+US exhibited significant damage to their cell walls and membranes, characterized by pronounced wrinkling and surface distortion (Figure 3f), attributable to the combined action of CO and ROS under US stimulation. These observations were further supported by TEM results, which revealed a loss of the distinct cellular profile in both pathogens, accompanied by a lighter cytoplasmic staining indicative of possible content leakage (Figure 3g). To assess the extent of membrane damage, bacteria were stained with the fluorescent probe N‐phenylnaphthalen‐1‐amine (NPN), with nucleic acids counterstained blue using Hoechst 33342, and NPN fluorescence visualized in red. Since NPN cannot penetrate an intact outer membrane, its dramatic fluorescence enhancement upon binding to the hydrophobic region of the phospholipid bilayer serves as a direct indicator of outer membrane damage [46]. Under US stimulation, the observed red fluorescence intensity increased progressively with the severity of damage, and visual assessment indicated that the 3HF@COFQDs incubation group exhibited stronger red fluorescence than the COFQDs group (Figure 3h). We subsequently investigated protein leakage by measuring the extracellular optical density at 280 nm (OD_280_), finding that the COFQDs+US treatment group showed higher leakage than the control, with the 3HF@COFQDs+US group demonstrating the most significant effect (Figure 3i). A subsequent membrane damage kinetics assay using the NPN probe confirmed this trend, with the 3HF@COFQDs+US treatment again showing the most pronounced kinetic profile (Figure 3j). In conclusion, ROS can directly disrupt the bacterial outer membrane, leading to protein leakage, while CO likely enhances the overall bactericidal effect by exacerbating metabolic dysfunction within the bacteria, thereby rendering them more vulnerable to ROS‐mediated attack.

Bacterial biofilms, known for their intrinsic resistance to both antibiotics and the host immune system, are notoriously difficult to eradicate and can lead to a variety of chronic bacterial infections [3]. To evaluate the inhibitory effect of 3HF@COFQDs on MRSA biofilm formation, we performed crystal violet staining. Optical microscopy images revealed distinct color shifts from violet to pale violet, indicating significant disruption of biofilm integrity (Figure 3k). Corresponding photographic evidence demonstrated that the 3HF@COFQDs+US group exhibited remarkable antibiofilm activity (Figure S13). In contrast, biofilms remained largely intact across all treatment groups not subjected to US stimulation. Quantitative analysis of crystal violet staining further confirmed that the 3HF@COFQDs+US group resulted in the lowest biofilm biomass compared to all other experimental conditions (Figure 3m). Subsequently, we conducted 3D reconstruction of live MRSA biofilms stained with SYTO 9 green fluorescent dye to visualize structural changes following different treatments (Figure 3l). Similarly, following treatment with 3HF@COFQDs+US, the biofilm architecture was severely disrupted, exhibiting thin and dispersed morphological features with only a few scattered bacterial colonies remaining. These results clearly indicate that the combination of 3HF@COFQDs with US effectively disrupts bacterial biofilms, attributable to the US‐triggered generation of CO and ROS, thereby confirming its efficacy in biofilm eradication.

### 2.4 In Situ Construction of the CO Depot

**FIGURE 4:**
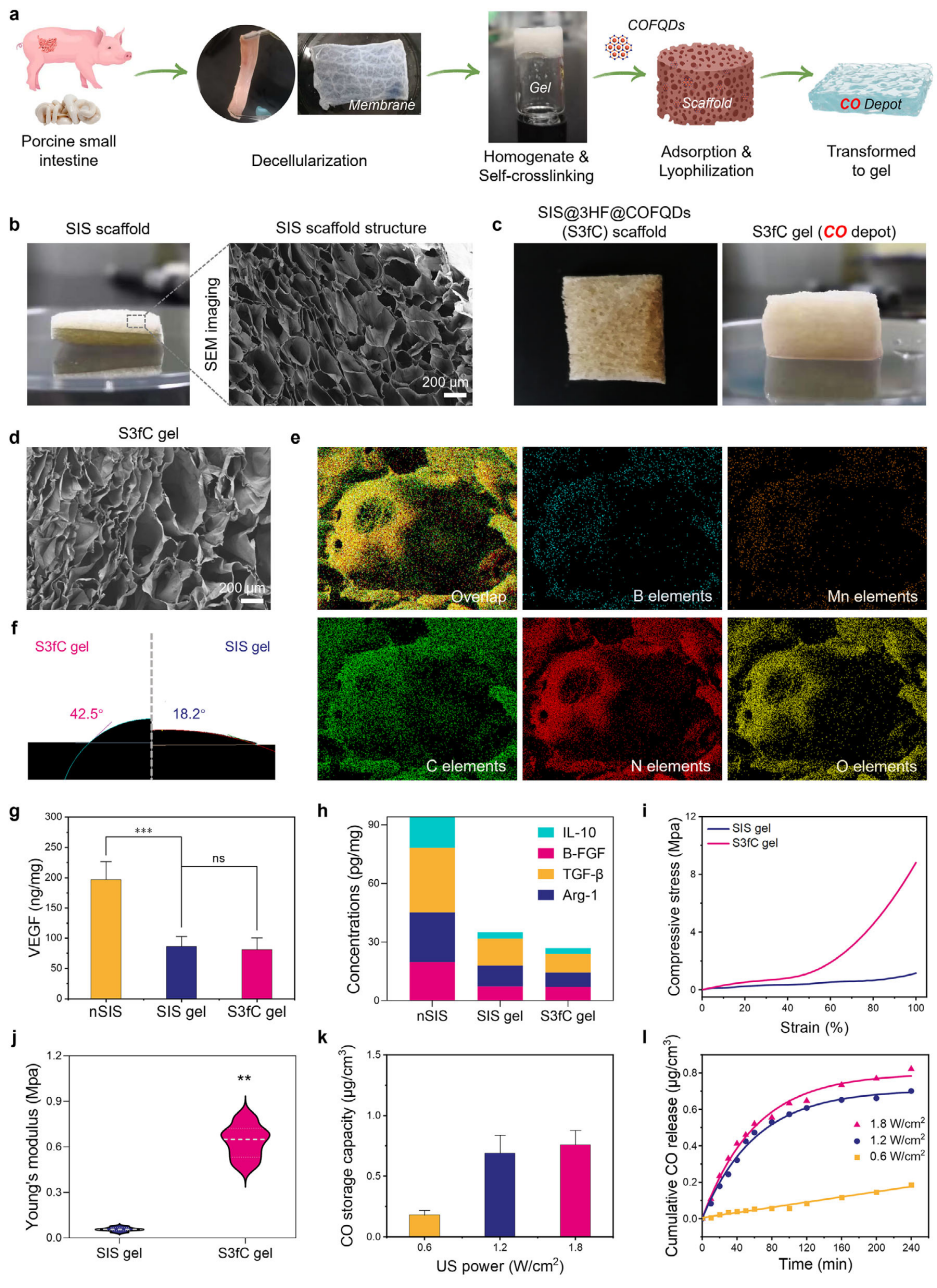
Fabrication and characterization of the US‐responsive CO depot. (a) Fabrication flowchart of the CO depot, involving decellularization, homogenization, and 3HF@COFQDs adsorption. (b) Digital photographs of the SIS scaffold and corresponding SEM images at higher magnification. (c) Digital photographs of the S3fC scaffold and gel (named CO depot). (d) Representative SEM image of S3fC gel. (e) SEM mapping images of C, N, O, B, and Mn elements in selected areas of S3fC gel. (f) Water contact angles of SIS gel and S3fC gel. (g) Levels of VEGF factor in nSIS, SIS gel, and S3fC gel. n = 3. (h) Growth factor content (IL‐10, b‐FGF, TGF‐β, and Arg‐1) in nSIS, SIS gel, and S3fC gel. (i, j) Compressive stress and Young's modulus in SIS gel and S3fC gel. n = 3. (k) Maximum CO storage capacity in S3fC gel under varying US power. n = 3. (l) CO release profile over time from S3fC gel following 30‐min US exposure at different powers in a 0.25 mg/mL collagenase buffer. Data are expressed as mean ± SD. Statistical significance was determined using a two‐sided *t*‐test, * denotes *p* < 0.05, ** denotes *p* < 0.01, *** denotes *p* < 0.001.

In severely infected tissues, the transient release of CO can inhibit pathogen growth, yet it remains insufficient to resolve persistent inflammation. Therefore, constructing a depot capable of sustained CO release is crucial for promoting post‑infection repair. Embedding 3HF@COFQDs into natural decellularized ECM polymers (such as SIS) represents an effective strategy. This approach preserves native biochemical repair components while enabling sustained therapeutic efficacy of CO at pathological sites. Moreover, the hydroxyl and diol groups on the COFQD surface can form hydrogen bonds with the concentrated SIS network to enhance mechanical properties [47]. The fresh porcine small intestine harvested from healthy pigs was decellularized through a sequential process involving mechanical separation, degreasing, enzymatic digestion, lyophilization, and sterilization to yield decellularized SIS. Subsequently, the prepared SIS scaffolds were immersed in an aqueous solution to yield SIS@3HF@COFQDs (named S3fC) gel by an adsorption process (Figure 4a). Finally, we added COFQDs to the SIS gel to obtain SIS@COFQDs (designated as SC gel) as a CO‑depleted control group.

To evaluate the decellularization efficiency of the SIS, hematoxylin and eosin (H&E) staining precisely revealed that the decellularized SIS exhibited an absence of cells and only trace amounts of cellular debris, whereas the porcine native SIS (nSIS) clearly displayed intact, large areas of cellular distribution (Figure S14). The DNA content of the decellularized SIS gel decreased by nearly two orders of magnitude compared to that of nSIS (Figure S15a), with its residual DNA content falling below 50 ng/mg, indicating that SIS could avoid adverse host responses in recipients. Moreover, following the decellularization process, the SIS gel retained approximately 50% of its glycosaminoglycan (GAG) and collagen levels (Figure S15b,c). This preservation creates a favorable microenvironment for tissue repair and regeneration, thereby facilitating the coagulation, proliferation, and migration processes in infected tissues. The prepared SIS scaffold exhibited a macroscopically visible sponge‐like porous structure and appeared nearly pure white (Figure 4b). SEM observation confirmed that the morphology of the SIS scaffold was as expected, mainly composed of uniformly distributed pores with sizes between 200 and 300 µm. After absorbing the nanoprecursors, the S3fC scaffold took on the brown color characteristic of 3HF@COFQDs, indicating maximum‐capacity (395.2 mg/g) adsorption of the material (Figure 4c). SEM imaging revealed that these ultrafine particles did not affect the inherent macroporous structure of the SIS (Figure 4d). Uniform distribution of B and Mn from 3HF@COFQDs was further observed via SEM equipped with EDX, which also verified the successful adsorption of 3HF@COFQDs (Figure 4e). After the loading of hydrophobic 3HF@COFQDs, the hydrophilicity of the S3fC gel decreased, and its water contact angle increased from 18.2° to 42.5° (Figure 4f). Therefore, the above results confirm the successful preparation of the S3fC gel, which serves as an ideal CO depot.

To evaluate changes in growth factor content during the multi‐step preparation of the S3fC gel, the concentrations of various key growth factors were analyzed using enzyme‐linked immunosorbent assay (ELISA). The data showed that the levels of VEGF, TGF‐β, interleukin 10 (IL‐10), basic fibroblast growth factor (b‐FGF), arginase 1 (Arg‐1), GAG, and collagen decreased significantly after decellularization of the SIS gel. After adsorption with 3HF@COFQDs, it did not further decrease (Figure 4g and Figure S15). At the final stage of preparing the S3fC gel (CO depot), the measured contents of VEGF, TGF‐β, IL‐10, b‐FGF, and Arg‐1 were 81.32 ng/mg, 2.97 pg/mg, 7.15 pg/mg, 9.60 pg/mg, and 7.21 pg/mg, respectively (Figure 4h). Furthermore, we examined changes in the mechanical properties of the SIS gel and the S3fC gel. The compressive stress of the S3fC gel was higher than that of the SIS gel, with compressive modulus and Young's modulus reaching 3.96 and 0.66 MPa, respectively (Figure 4i,j). This enhancement may be attributed to the multi‑hydroxy hydrogen‑bonding effects on the surface of the COFQDs [48]. The storage and controlled release capacity of the CO depot are crucial for anti‑infection therapy. We therefore assessed its maximum CO‑loading capacity under US stimulation. Samples were irradiated in a sealed container with US at different power densities, followed by acid‑induced cleavage of the COFQD framework. The results indicated that CO loading at 1.2 and 1.8 W/cm^2^ was similar, reaching approximately 0.7 µg/cm^3^, which was substantially higher than that obtained at 0.6 W/cm^2^ (Figure 4k). Next, the controlled‑release behavior of the CO depot under different US power densities was evaluated. Over a period of 240 min, irradiation at 1.2 and 1.8 W/cm^2^ enabled slow release of CO gas, thereby avoiding additional toxicity associated with excessive CO exposure (Figure 4l). Due to the barrier effect posed by the flexible network of the SIS gel layer, the CO release time was doubled compared to that of 3HF@COFQDs (Figure 2j). The naturally derived SIS is prone to degradation under physiological conditions, leading to the disintegration of the flexible shell of the S3fC gel and the gradual exposure of 3HF@COFQDs, thereby enabling flexible gate‐controlled release. We evaluated the degradation rate of the S3fC gel and found that at 120 h, the gel had degraded by 95.4%, which coincided with the duration of sustained CO release (Figure S16a,b). Based on the findings above, which indicated little difference in CO generation and release between 1.2 and 1.8 W/cm^2^ US, we selected 1.2 W/cm^2^ US for the subsequent experimental workflow. Next, we measured the antibacterial activity of the CO depot under sustained‐release conditions. The unmodified SIS gel showed no inherent antimicrobial activity, while the SC gel treated with US exhibited a power‑dependent antibacterial activity (Figure S17). Following the introduction of CO, the S3fC+US group exhibited the lowest MRSA load, further underscoring the critical role of CO in synergistically enhancing antibacterial activity. In summary, our results confirm the hypothesis regarding the in situ construction of US‐responsive CO depots, which can be further applied to early treatment for severe infections.

### 2.5 Anti‐Inflammatory and Immunological Effects of CO Depots

**FIGURE 5:**
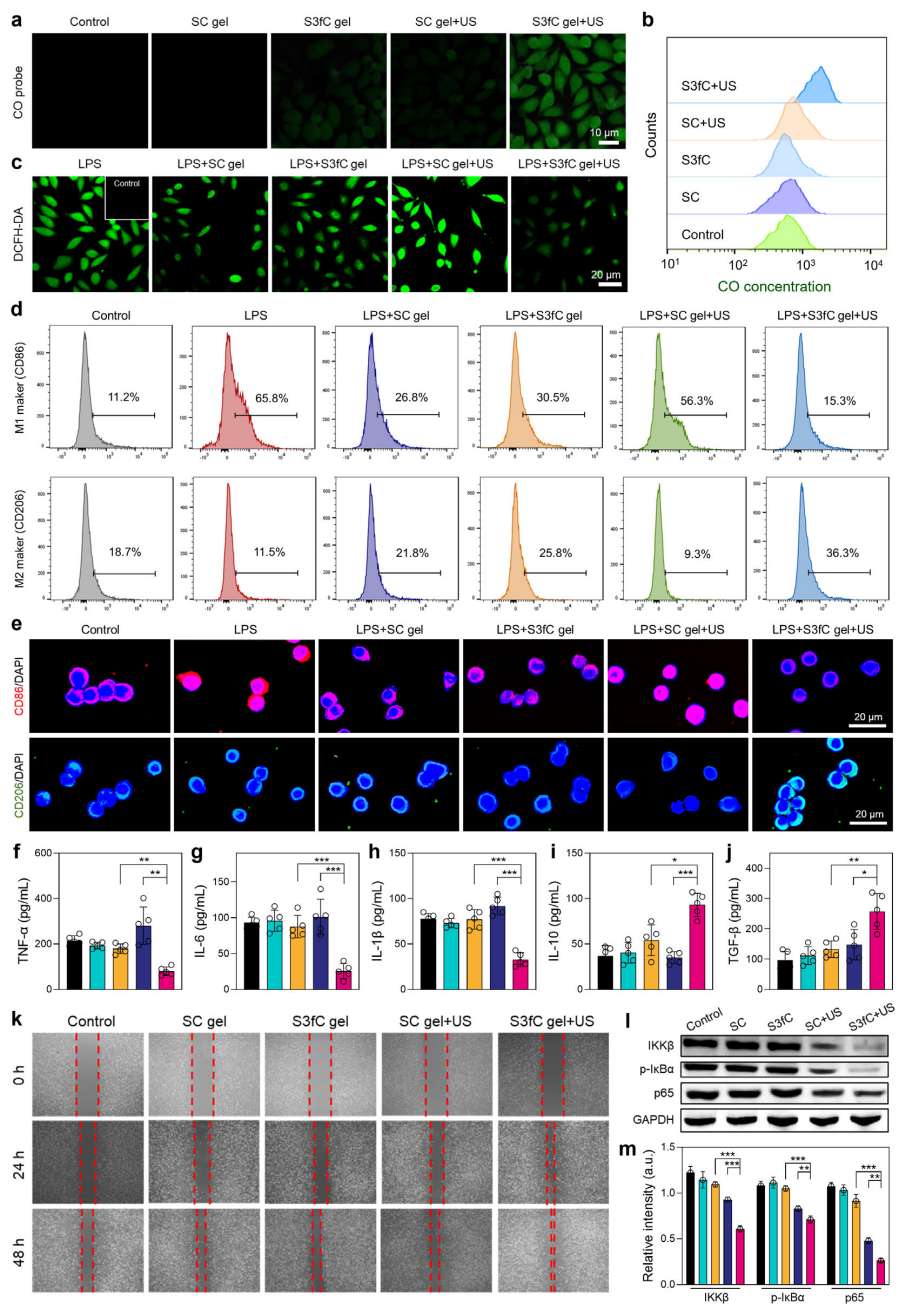
Cellular and molecular analysis of the therapeutic effects of CO depots. (a) Detection of CO release in HUVECs following different treatments using a CO fluorescent probe. (b) Flow cytometric analysis of endogenous CO levels in macrophages. (c) Fluorescence images of HUVECs with ROS stained by DCFH‐DA probe treated by PBS (negative control), LPS (positive control), LPS+SC gel, LPS+S3fC, LPS+SC gel+US, and LPS+S3fC+US. (d) Flow cytometry analysis of macrophage biomarkers CD86 (M1) and CD206 (M2). (e) Analysis of the expression levels of CD86 and CD206 in macrophages by immunofluorescence staining. (f‐j) Levels of pro‐inflammatory (TNF‐α, IL‐6, and IL‐1β) and anti‐inflammatory (IL‐10 and TGF‐β) cytokines secreted by macrophages following treatment with PBS, SC gel (with or without US), and S3fC gel (with or without US). n = 5. (k) Cell scratch migration assay and migration effects of different treatment groups at 24 and 48 h. (l, m) Western blotting and corresponding quantification for IKKβ, p‐IκBα, and p65 expression in macrophages following different treatments. n = 3. Data are expressed as mean ± SD. Statistical significance was determined using a two‐sided *t*‐test, * denotes *p* < 0.05, ** denotes *p* < 0.01, *** denotes *p* < 0.001.

After constructing the CO depots, we thoroughly investigated the therapeutic effects of the CO and SIS combination on inflamed tissues through in vitro cellular experiments, systematically evaluating their regulatory impact on simulated inflammatory microenvironments and their ability to modulate immune cell behavior. CO primarily enters cells via passive diffusion and modulates downstream signaling pathways [11, 49]. First, we used the CO fluorescent probe 1 to detect intracellular CO levels in endothelial cells. Regardless of the presence or absence of US irradiation, the SC gel did not produce CO (Figure 5a and Figure S18a). In contrast, under S3fC+US conditions, intracellular CO levels were significantly higher than those of S3fC without US irradiation, indicating that the CO depots can effectively deliver CO into cells. To further evaluate CO delivery to immune cells, intracellular CO levels in macrophages were quantified by flow cytometry. The S3fC gel+US group exhibited a significantly enhanced intracellular CO signal, confirming effective CO delivery to macrophages (Figure 5b). Next, we examined the effect of the S3fC gel on ROS levels in inflamed cells. An in vitro inflammation model was established by stimulating human umbilical vein endothelial cells (HUVECs) with lipopolysaccharide (LPS). In the absence of US, the ROS fluorescence intensity in both the SC gel and S3fC gel groups was significantly higher than that in the control group but lower than that induced by LPS stimulation alone; this reduction might be attributed to the effects of SIS (Figure 5c and Figure S18b). Notably, after SC gel+US treatment, ROS levels in HUVECs increased markedly, which may be attributed to additional ROS generated by COFQDs under US exposure. However, with CO intervention, the S3fC gel+US group exhibited a substantial decrease in ROS, demonstrating that CO can suppress ROS storm in inflammatory cells.

Macrophages play a crucial role in the early stages of inflammatory response and tissue repair following microbial infection [50]. Therefore, it is essential to evaluate whether CO depots can interact with immune cells such as macrophages. To this end, we assessed the polarization of RAW 264.7 cells using confocal laser scanning microscopy (CLSM) and flow cytometry. CD86 is recognized as a surface marker for M1 macrophages, while CD206 is commonly used to identify M2 macrophages [50]. Flow cytometry analysis revealed that neither CD86 (M1 marker) nor CD206 (M2 marker) was prominently expressed in untreated macrophages, indicating that most cells remained in a quiescent state. After LPS stimulation, CD86 expression became dominant, confirming polarization to the M1 phenotype. When LPS‑stimulated macrophages were co‑cultured with SC gel+US, they exhibited increased CD86 signal and decreased CD206 signal, attributable to US‐induced ROS generation that elevated the inflammatory state. (Figure 5d). Notably, the protein expression of CD86 in the S3fC gel+US group was significantly lower than in the LPS and SC gel+US groups, whereas CD206 expression was markedly higher. These findings align with immunofluorescence staining results, which showed the lowest CD86 intensity and the highest CD206 intensity in the S3fC gel+US group (Figure 5e). Protein expression of representative M1 markers (TNF‑α, IL‐6, and IL‑1β) and M2 markers (IL‑10 and TGF‑β) was further examined via ELISA kits. In LPS‑stimulated macrophages, expression of proinflammatory cytokines was significantly upregulated. Excess ROS generated by the SC gel+US treatment promoted a pro‐inflammatory macrophage response, characterized by upregulation of TNF‐α, IL‐6, and IL‐1β (Figure 5f‐h) and downregulation of IL‐10 (Figure 5i,j). Compared with SC gel+US, S3fC gel+US induced lower expression of IL‑1β, TNF‑α, and iNOS, along with higher expression of IL‑10 and TGF‑β. These results suggest that the US‑responsive rigid depot and controlled release of CO may serve as an effective regulator driving macrophage transition from the M1 to the M2 phenotype.

Based on the anti‐inflammatory effects of CO depots with US irradiation, a series of assays, including CCK‐8 proliferation, Transwell migration, tube formation, and scratch wound healing, were performed to investigate the effects of CO depots on cells involved in tissue regeneration following infection. The results showed that at a concentration of 200 µg/mL, none of the materials except COFs exhibited cytotoxicity, while COFQDs and 3HF@COFQDs did not show any cytotoxic effects (Figure S19). Interestingly, SIS gel, SC gel, and S3fC gel all promoted substantial cell proliferation. The results of the Transwell assay were consistent with the conclusions drawn from the cytotoxicity experiment, further proving that the CO depot indeed induces cell proliferation (Figure S20). In addition, CLSM staining was used to label cellular metabolic activity, revealing that the S3fC gel+US treatment group exhibited the highest fluorescence intensity, surpassing both the SC gel, SC gel+US, and S3fC gel groups (Figure S21). The scratch wound healing assay demonstrated that the healing rate in the S3fC gel+US treatment group increased by approximately 19% and 18% at 24 h, respectively, compared to other control groups, providing direct visual evidence of its pro‐migratory effect (Figure 5k and Figure S22). We co‐cultured SIS gel, SC gel, and S3fC gel with red blood cells to observe their impact on erythrocytes. All three gels showed extremely low hemolysis rates (<3%), laying the foundation for subsequent blood‐contact anti‐infection applications in vivo (Figure S23).

Subsequently, we employed Western blotting to analyze the potential mechanism behind the anti‐inflammatory effects of CO. The NF‐κB signaling pathway is recognized as a central and ubiquitous way of inflammatory responses [51]. We first analyzed the inhibition/activation status of this pathway. Upon extracellular stimulation, the IκB kinase (IKK) complex (including IKKβ) is activated and subsequently phosphorylates IκBα to generate p‐IκBα, which is then tagged for degradation, thereby releasing NF‐κB for nuclear translocation and pathway activation [52]. p65 recruits activating complexes to the promoters of target genes, enhancing the transcription of inflammatory genes [53]. We found that IKKβ and phosphorylated inhibitor of nuclear factor kappa B alpha (p‐IκBα) levels were significantly downregulated in the S3fC+US group, indicating that both the upstream activation signal and the downstream degradation process in the NF‐κB pathway are inhibited (Figure 5l,m). Notably, the expression of p65 also significantly decreased after CO depot treatment, suggesting a marked decline in the transcription efficiency of NF‐κB target genes, and that cellular inflammation could not be effectively initiated. In summary, the US‐triggered CO depot can restore inflamed cells to a normal state, reduce intracellular oxidative stress levels, and induce M2 polarization in macrophages, potentially through the NF‐κB pathway.

### 2.6 RNA‐seq of Inflamed Macrophages

**FIGURE 6:**
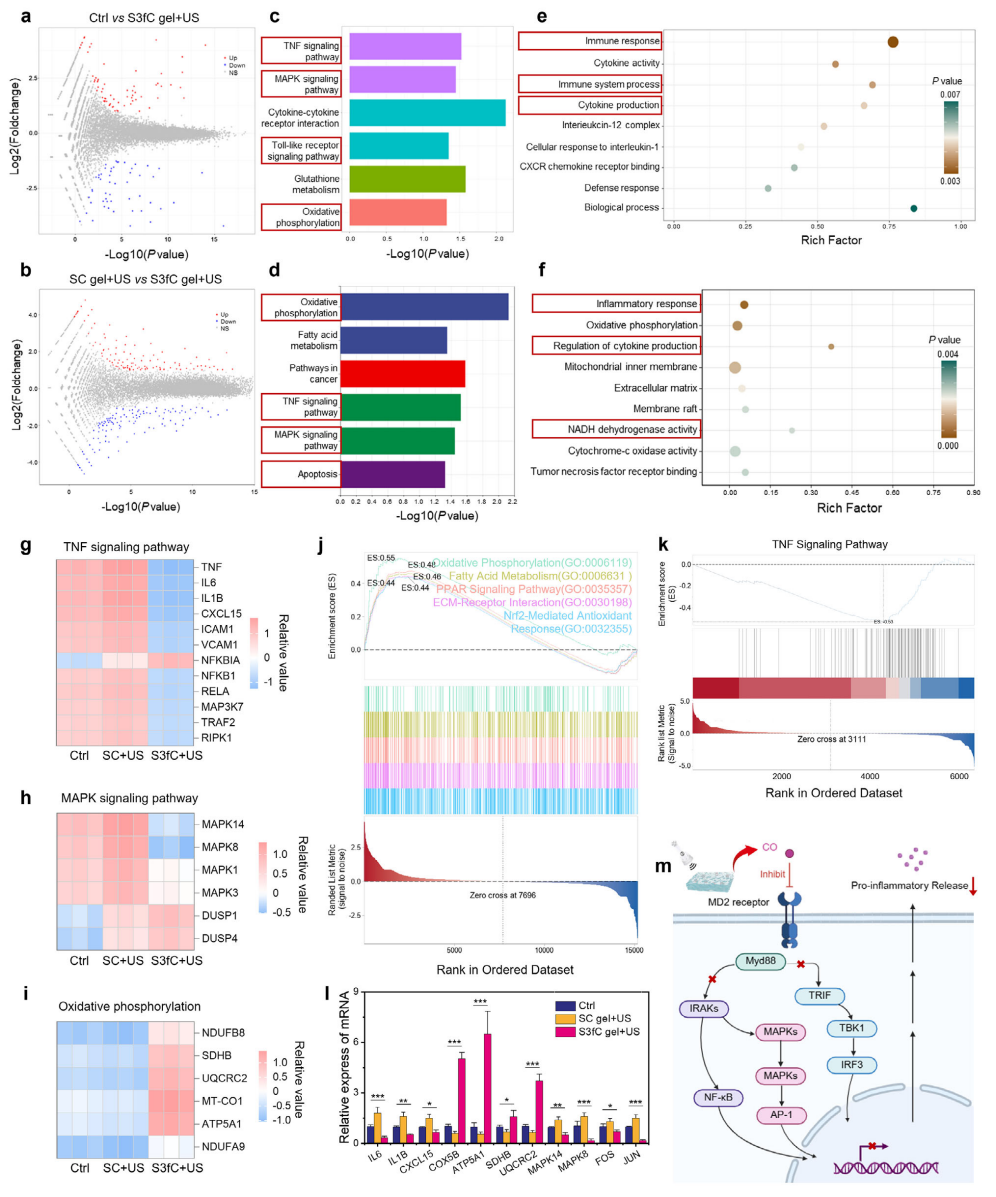
Transcriptomic analysis of therapeutic modulation in inflamed macrophages. (a, b) Volcano plots for the DEG distribution in the S3fC gel+US group compared with Control and S3fC gel+US group compared with SC gel+US. (c, d) KEGG enrichment analysis of up‐ and down‐regulated pathways in S3fC gel+US versus Control and S3fC gel+US versus SC gel+US. (e, f) GO enrichment analysis of up‐ and down‐regulated pathways in S3fC gel+US versus Control and S3fC gel+US versus SC gel+US. (g‐i) Heatmaps of DEGs involved in the TNF signaling, MAPK signaling, and oxidative phosphorylation pathways between the Control, SC gel+US, and S3fC gel+US groups. (j, k) GSEA enrichment analysis of up‐ and down‐regulated pathways in S3fC gel+US versus Control. (l) The qPCR results of the representative genes involved in TNF signaling, MAPK signaling, and oxidative phosphorylation pathways. (m) Schematic illustration of the anti‐inflammatory mechanism mediated by CO depot based on transcriptomic analysis. Data are expressed as mean ± SD; n = 5. Statistical significance was determined using a two‐sided *t*‐test, * denotes *p* < 0.05, ** denotes *p* < 0.01, *** denotes *p* < 0.001.

As mentioned above, the S3fC gel demonstrated potent antibacterial and anti‑inflammatory activities both in vitro and in vivo. To systematically elucidate the molecular mechanism and to isolate the specific contribution of CO, we performed RNA‑seq on macrophages under three conditions: Control, S3fC gel+US, and SC gel+US (without CO generation). We conducted two comparisons: S3fC gel+US vs Control to identify overall changes, and S3fC gel+US vs SC gel+US to assess the effect of CO. Volcano plots showed distinct gene expression patterns in both comparisons, with NF‑κB/MAPK‑related genes prominently downregulated in the S3fC gel+US group relative to both Control and SC gel+US (Figure 6a,b). KEGG pathway enrichment of S3fC gel+US vs Control highlighted TNF, MAPK, Toll‑like receptor, and oxidative phosphorylation pathways (Figure 6c,d). Critically, the S3fC gel+US vs SC gel+US comparison confirmed that these pathways were significantly downregulated due to CO. GO enrichment of S3fC gel+US vs Control showed regulation of immune response and cytokine production (Figure 6e,f), and the direct comparison with SC gel+US gave consistent results.

Heatmaps of key pathways further illustrated the specific effect of CO. Notably, the expression patterns of the SC gel+US group were similar to those of the Control group (Figure 6g‐i). In the S3fC gel+US vs Control comparison, TNF pathway genes (*TNF*, *IL‑6*, *IL‑1β*, *CXCL15*) were downregulated (Figure 6g); mitogen‐activated protein kinase (MAPK) pathway genes (*MAPK14*, *MAPK8*, *MAPK1*, *MAPK3*) were downregulated while DUSP1/4 were upregulated (Figure 6h); Oxidative phosphorylation (OXPHOS) genes (*NDUFB8*, *SDHB*, *UQCRC2*, *MT‑CO1*) were upregulated (Figure 6i). The S3fC gel+US vs SC gel+US comparison confirmed that the same gene sets were similarly altered, with downregulation of pro‑inflammatory genes and upregulation of OXPHOS genes specifically in the presence of CO. Toll‑like receptor pathway genes (*Ifnb1*, *Cxcl10*, *Irf7*, *Isg15*) were downregulated in S3fC gel+US vs Control (Figure S24), and the direct comparison with SC gel+US confirmed CO dependence [54]. Gene Set Enrichment Analysis (GSEA) of S3fC gel+US vs Control showed enrichment of OXPHOS, fatty acid metabolism, PPAR signaling, ECM‑receptor interaction, and Nrf2 response, while TNFA/NF‑κB signaling was downregulated (Figure 6j,k). Quantitative polymerase chain reaction (qPCR) validation of representative genes (*IL‑6*, *IL‑1β*, *CXCL8*, *COX5B*, *ATP5A1*, *SDHB*, *UQCRC2*, *MAPK14*, *MAPK8*, *FOS*, *JUN*) was consistent for both comparisons, confirming that these changes were specifically driven by CO release (Figure 6l, Table S1).

Under US triggering, the S3fC gel gradually transforms into a CO depot, releasing CO that has been reported to inhibit the NF‐κB pathway [55]. We hypothesize that CO binds to the membrane receptor MD2, suppresses a cascade of kinases and transcription factors, blocks the NF‐κB/MAPK/IRF3 axis, and downregulates pro‐inflammatory factors such as TNF‐α and IL‐6 (Figure 6m). Together, these results demonstrate that the anti‑inflammatory transcriptional signature is specifically attributable to released CO, not to US or the carrier alone, supporting the therapeutic potential of this platform.

### 2.7 In Vivo Evaluation of Acute Infection

**FIGURE 7:**
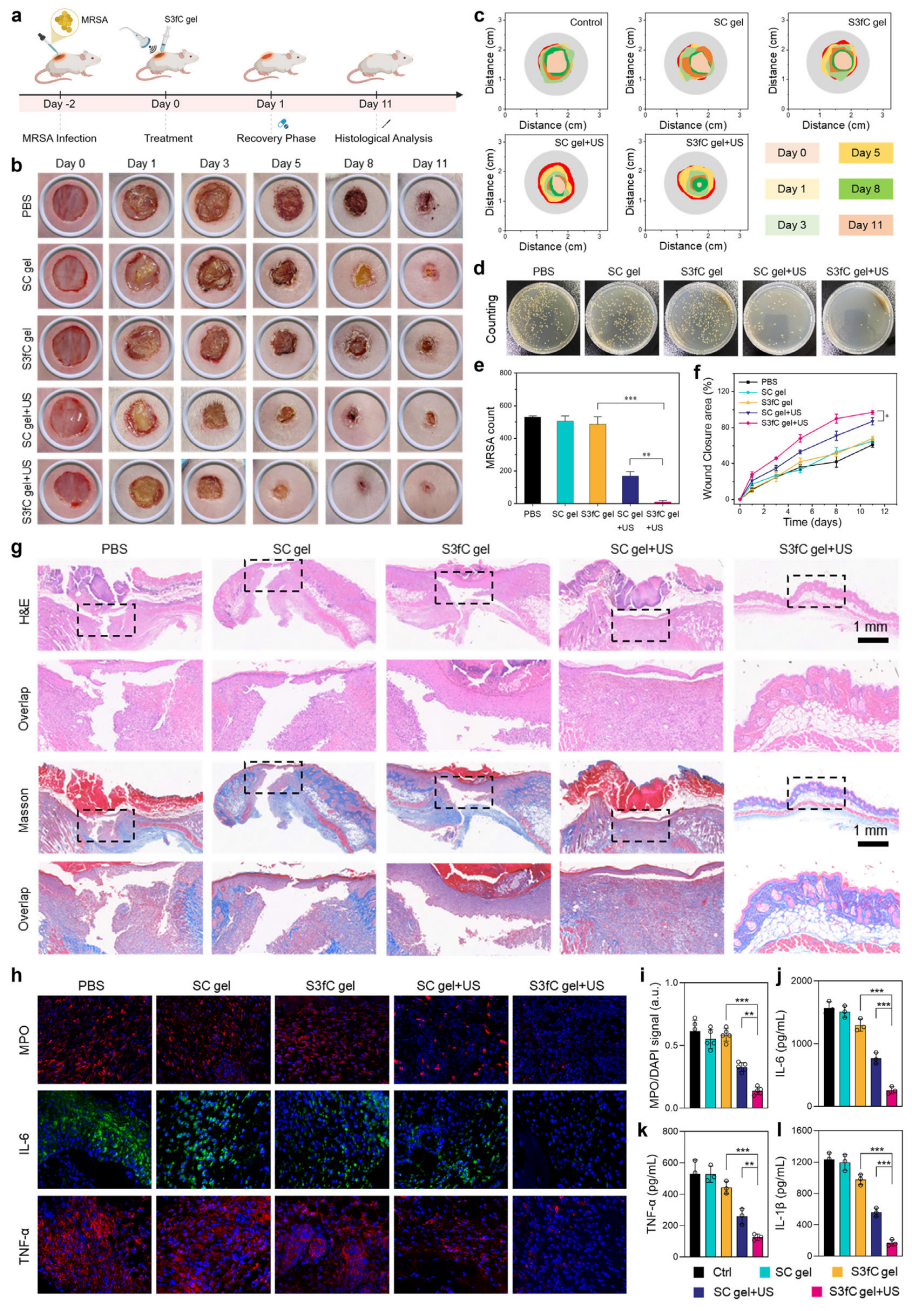
Comprehensive evaluation of treatment efficacy in an acute infection model. (a) Schematic of the CO depot treatment regimen for MRSA acute infected wound model. (b) Photographs of infected mice wounds treated with PBS only, SC gel, S3fC gel, SC gel+US, and S3fC gel+US, respectively. (c) Schematic diagram showing wound traces for each group at various time points. (d) MRSA colony counts around the infected wounds through the spread plate method. (e) Statistical data of MRSA burden in wounds from various treatment groups on day 11. n = 5. (f) Quantitative analysis of the residual wound areas from different treatment groups. n = 5. (g) H&E staining and Masson staining of the infected skin slices after different treatments on day 11. (h) Immunofluorescence images of MPO, IL‐6, and TNF‐α staining of infected sites after different treatments. (i) Quantification of the mean fluorescence intensity for the areas positive for MPO/DAPI signal. n = 5. (j‐l) ELISA analysis of the expression levels of IL‐6, TNF‐α, and IL‐1β in acute wound tissues following different treatments. n = 3. Data are expressed as mean ± SD. Statistical significance was determined using a two‐sided *t*‐test, * denotes *p* < 0.05, ** denotes *p* < 0.01, *** denotes *p* < 0.001.

After confirming the antibacterial activity in vitro, we proceeded to assess the therapeutic efficacy of a US‐mediated CO depot in treating acute infected wounds. A full‐thickness wound model of approximately 10 mm was created on the backs of BALB/c mice using surgical scissors, and the wounds were infected by applying 100 µL of logarithmic‐phase MRSA (1×10^8^ CFU/mL). The infected mice were randomly divided into five groups (n = 5): (I) Control, (II) SC gel, (III) S3fC gel, (IV) SC gel+US, and (V) S3fC gel+US. The mice were then treated accordingly on intervening days, and wound sizes were recorded over 11 days (Figure 7a). Concurrently, the body weight of the mice in all groups remained stable throughout the treatment period (Figure S25). Following MRSA colonization on day 0, apparent yellowish pathogen adhesion signs were observed on the wound surface (Figure 7b). The wounds treated with PBS, SC gel, and S3fC gel followed a relatively slow healing process, with large wound areas on day 11. As expected, wounds in the S3fC gel+US group were almost completely healed on day 11, significantly outperforming the SC gel+US group. This enhanced efficacy is attributable to the sustained release of CO under US activation. Wound area sizes were simulated using software for clearer visualization and comparison, revealing that wounds treated with S3fC gel+US exhibited significantly reduced dimensions (Figure 7c). To further evaluate the ablation effect of the CO depot on MRSA in the acute infection model, the number of residual bacteria on infected wounds was quantified by colony counting (Figure 7d). The experimental results further demonstrated that S3fC gel combined with efficient US irradiation exhibited excellent antibacterial properties, with only 0.2% viable bacteria remaining on LB plates on day 3. In contrast, groups SC gel, S3fC gel, and SC gel+US retained approximately 95.3%, 91.9%, and 31.9% viable bacteria, respectively (Figure 7e). This was consistent with the wound closure trend: S3fC gel+US group showed the strongest wound‐healing promotion in biofilm wounds, with a wound closure rate of approximately 97.36% on day 11, exceeding that of the other groups (Figure 7f).

On day 11, mice from each experimental group were euthanized, and wound tissues were collected for subsequent histological analysis, including H&E staining and Masson's trichrome staining. Granulation tissue in the PBS, SC gel, and S3fC gel groups appeared loose and discontinuous (Figure 7g). SC gel+US and S3fC gel+US groups exhibited more abundant granulation tissue regeneration (Figure 7g). Following combined treatment, dense and multilayered granulation tissue formed at the wound site, with tissue thickness being significantly greater in the S3fC gel+US group than in other groups. Fibroblast proliferation was markedly enhanced in the wound tissues of the S3fC gel+US group, likely due to the synergistic effect of the CO depot promoting cell migration and thereby facilitating tissue remodeling. Masson's trichrome staining also reflected collagen deposition within the granulation tissue. Collagen deposition was very thin and disorganized in PBS, SC gel, and S3fC gel groups. In contrast, collagen deposition in the regenerated wounds of SC gel+US and S3fC gel+US groups showed a certain degree of alignment. Notably, the S3fC gel+US group displayed the most orderly arrangement and the highest expression of collagen fibers (Figure S26). Wound healing was further evaluated through comprehensive histological assessment, including examination of inflammatory cell infiltration and cytokine indicators. PBS, SC gel, and S3fC gel groups all exhibited varying degrees of acute inflammation (Figure S27), attributed to the migration of inflammatory cells such as macrophages and monocytes to the wound area to restore microenvironmental homeostasis. In contrast, due to the timely and sustained release of CO upon S3fC gel+US stimulation, wound tissues in the S3fC gel+US group showed relatively mild inflammation, with reduced inflammatory cell aggregation, indicating significantly attenuated inflammatory activity and resulting in scar tissue being replaced by newly formed epithelial tissue. Myeloperoxidase (MPO) is integral to neutrophil extracellular traps (NETs) for amplifying inflammatory responses, which are composed of proteins such as neutrophil elastase, cathepsin G, and histones [56]. The S3fC gel+US group exhibited the lowest immunofluorescence expression of MPO at the infected tissue site by CLSM, suggesting that CO combined with SIS may possess the potential to inhibit NETs formation (Figure 7h,i). The distribution of pro‐inflammatory cytokines downstream of the NF‐κB pathway was subsequently examined. In the absence of US stimulation, the expression levels of IL‐6 and TNF‐α remained nearly identical across groups. Following US intervention, both the SC gel and S3fC gel progressively downregulated the expression of these two cytokines, with the most pronounced reduction observed in the S3fC gel+US group. The expression of IL‑6, TNF‑α, and IL‑1β in tissues was analyzed using ELISA kits (Figure 7j‐l). The results align with the immunofluorescence staining data, showing that treatment with S3fCgel+US significantly reduced the levels of all three inflammatory factors. This suggests that the sustained‐release CO therapy can continuously supply carbon monoxide, thereby mitigating tissue inflammation caused by acute infection.

### 2.8 In Vivo Evaluation of Chronic Infection and Immunological Evaluation

**FIGURE 8:**
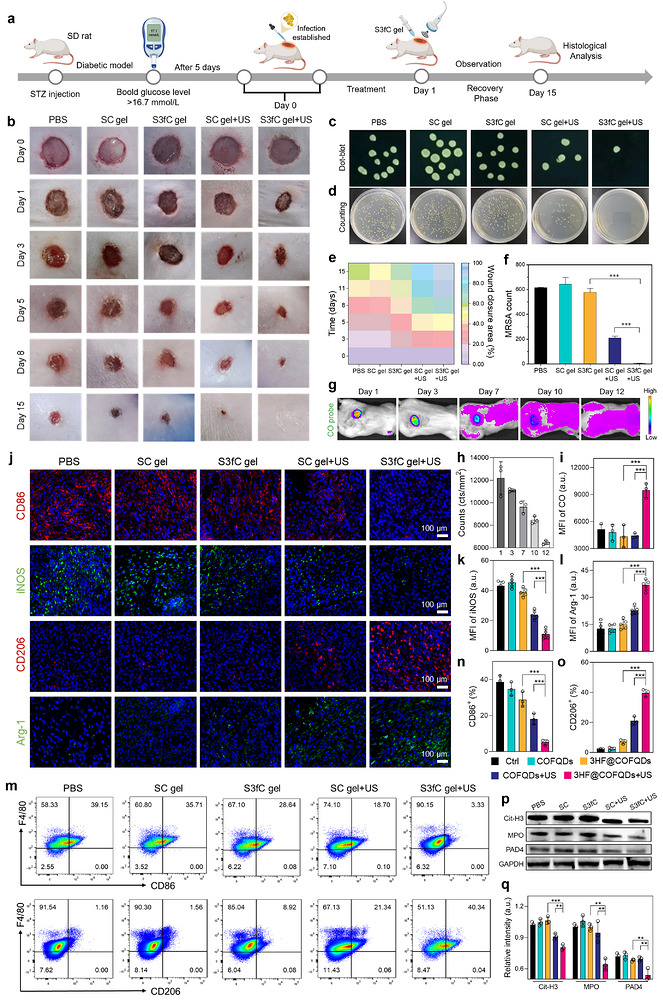
Evaluation of therapeutic response and associated immune reprogramming in infected wounds. (a) Schematic flowchart of the diabetic rat model establishment and the treatment regimen for the MRSA‐infected wound model. (b) Representative images of wound infection for each group at days 0, 1, 3, 5, 8, and 15. (c) The homogenate of wound tissues post‐treatment was spotted onto LB agar plates for spot assay. (d) Digital photographs of MRSA CFUs from treatment groups. (e) Heatmap displays the corresponding statistics of wound closure rates for different treatment groups at a specific time point. n = 5. (f) Statistical data of MRSA CFU in wounds from various treatment groups on day 15. n = 5. (g, h) Quantification of CO fluorescence intensity in wound regions after various treatments. n = 3. (i) Quantification of the mean fluorescence intensity of CO in different treatment groups by flow cytometry. n = 3. (j‐l) Immunofluorescence images and corresponding quantification of CD86, iNOS, CD206, and Arg‐1 staining of lesion sites after different treatments on day 15. n = 5. (m‐o) Flow cytometry assay and corresponding quantification for macrophage cells after different treatments (F4/80^+^/CD86^+^ and F4/80^+^/CD206^+^). n = 3. (p, q) Western blotting and corresponding quantification for Cit‐H3, MPO, and PAD4 expression of different treatment groups. n = 3. Data are expressed as mean ± SD. Statistical significance was determined using a two‐sided *t*‐test, * denotes *p* < 0.05, ** denotes *p* < 0.01, *** denotes *p* < 0.001.

The repair of tissues after chronic infection poses a major clinical challenge, as pathogens often resist treatment through mechanisms like biofilm formation, while persistent inflammation disrupts the local microenvironment and impedes regeneration. To evaluate whether the CO depot could enable a programmatically regulated strategy for chronic wound therapy, we established a model of diabetic chronic wounds and detailed the overall experimental workflow of an MRSA‐infected chronic wound model (Figure 8a). Streptozotocin (STZ) was injected into Sprague‑Dawley rats to induce diabetes [57]. A full‑thickness skin wound (∼15 mm diameter) was created on the back of each rat, followed by injection of MRSA to induce infection. Infected rats were randomly divided into five groups receiving different treatments: PBS, SC gel, S3fC gel, SC gel+US, and S3fC gel+US. At predetermined time points, wounds were imaged, total bacterial load in the skin lesions was determined, and immunological assessments were performed. First, changes in the infected lesions across groups were recorded, and the final healed area was quantified (Figure 8b and Figure S28). By day 15, chronic wounds in the S3fC gel+US group were nearly completely closed, whereas wounds in the other groups remained visibly unhealed. Quantitative spot dilution and CFU analysis of different wound tissues after treatment revealed that bacterial burdens were significantly reduced in the SC gel and S3fC gel groups under US intervention. The S3fC gel+US group exhibited the lowest bacterial burden, which is attributable to the sustained release of CO. (Figure 8c,d). The quantification of the healing process and CFUs demonstrated that the S3fC gel+US group achieved the highest wound healing rate (100%) and the corresponding CFUs were 4±2, both significantly superior to those of the other groups (Figure 8e,f). Moreover, the average body weight of rats receiving S3fC gel combined with US remained within the normal range within 15 days after MRSA injection (Figure S29).

Next, we monitored the in vivo biodistribution of CO released from the CO depot in infected rats using live‐animal fluorescence imaging. To assess the retention of CO in infected tissue over time, a CO probe 1 system was intravenously injected on days 1, 2, 3, 5, and 7 to react with CO at the wound site and generate fluorescence. Significant fluorescence was maintained at the wound site for up to 10 days (Figure 8g,h), which is due to the S3fC gel functioning as an effective reservoir for CO, facilitating its storage and subsequent sustained release. Flow cytometry analysis confirmed that the detected CO originated specifically from the S3fC gel+US group (Figure 8i and Figure S30). These results demonstrate that the S3fC hydrogel enables sustained CO release at chronic wound sites under US stimulation, consistent with the findings from in vitro studies. To further quantify local CO levels, we measured CO concentrations in wound tissue using a CO‑sensitive electrode calibrated against GC [58]. In the S3fC gel+US group, the CO concentration reached 5.8 ± 1.1 µM at 2 h post‑activation and remained within 1.6–5.5 µM over the first 48 h (Figure S31). Given the critical role of CO in post‐infection tissue repair and immune microenvironment remodeling, we evaluated macrophage polarization in infected rats via immunofluorescence staining. Compared with controls, the S3fC gel+US group showed significantly decreased expression of CD86 and iNOS, alongside increased expression of CD206 and Arg‑1 (Figure 8j‐l and Figure S32). This shift, not observed in other groups, suggests a transition from M1 to M2 macrophage phenotype in the tissue. Flow cytometry quantified the proportions of each phenotype: among F4/80^+^ cells, the S3fC gel+US group had only 3.33% CD86^+^ cells (versus 39.15% in controls) and 40.34% CD206^+^ cells (versus 1.16% in controls) (Figure 8m‐o). The sustained release of CO ultimately promotes the transition of macrophages from the pro‐inflammatory M1 phenotype to the anti‐inflammatory M2 phenotype, thereby aiding in the treatment of chronic infections during the later stages. To examine other immune cell activities, we assessed the activation of NETs after treatment [59]. Western blot analysis revealed marked downregulation of Cit‑H3, MPO, and PAD4 proteins, confirming strong suppression of the NETs pathway in vivo and reduced tissue inflammation (Figure 8p,q). This helped establish a pro‑regenerative microenvironment that enhanced the healing of infected chronic wounds. Together, these results demonstrate that the CO depot exerts a broad anti‑infective effect, attributable to the combined roles of SIS and CO.

### 2.9 Post‐Infection Tissue Repair Mediated by CO Depot

**FIGURE 9:**
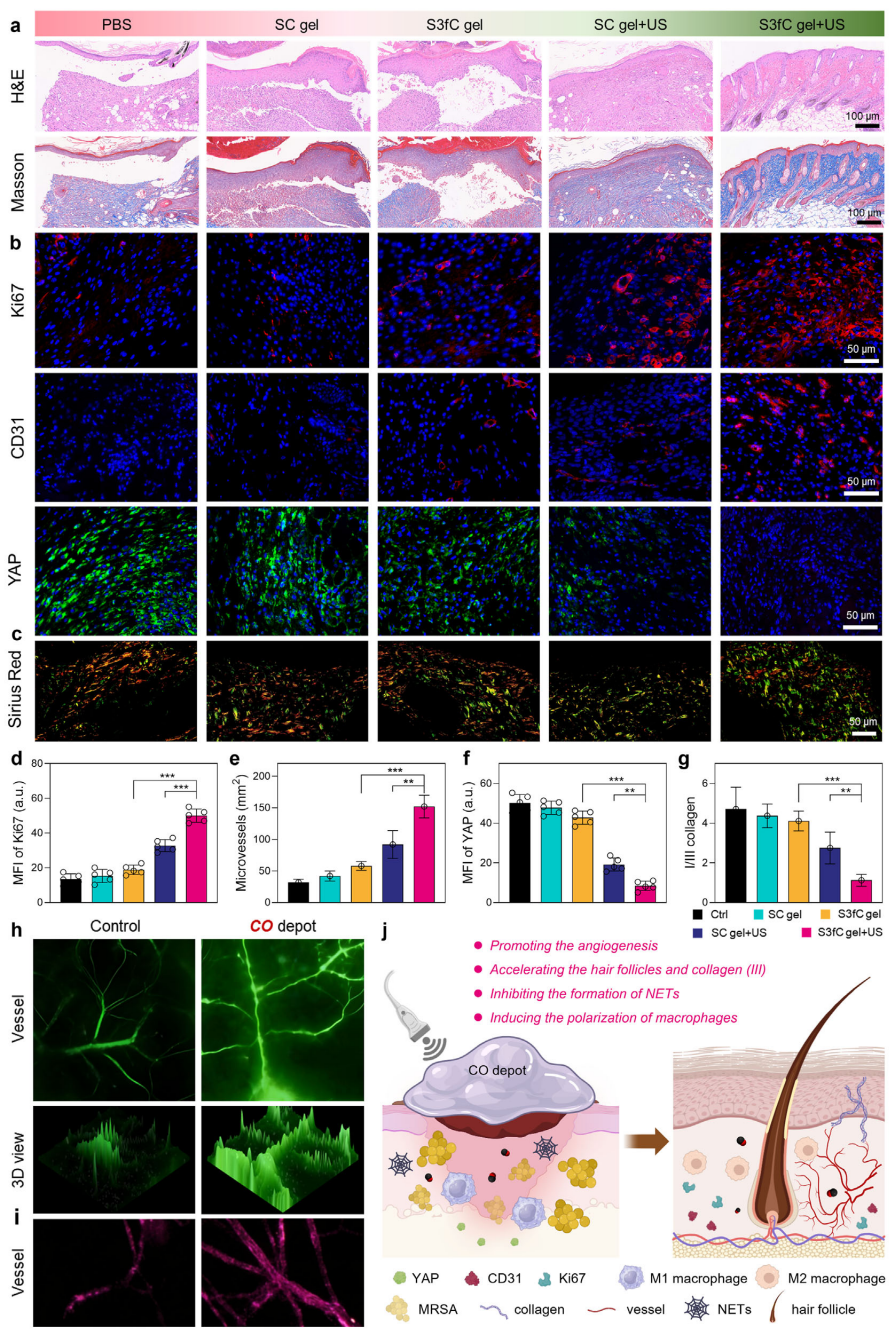
Comprehensive evaluation of tissue repair and vascular regeneration. (a) H&E staining and Masson staining of the infected skin slices on day 15. (b) Immunofluorescence images of Ki67, CD31, and YAP staining of lesion sites after different treatments on day 15. (c) Sirius Red staining of the infected skin slices on day 15. (d‐f) Quantification of the mean fluorescence intensity in Ki67, CD31, and YAP positive regions. (g) Ratio of type I/III collagen on day 15 after different treatment. (h) Near‐infrared fluorescence imaging and 3D views of vessels following PBS and CO depot treatment in infected rat tissue. (i) Near‐infrared vessel imaging of α‐SMA following PBS and CO depot treatment in infected rat tissue. (j) Graphical summary of the proposed processes and advantages underlying tissue repair following CO depot treatment in infected tissues. Data are expressed as mean ± SD; n = 5. Statistical significance was determined using a two‐sided *t*‐test, * denotes *p* < 0.05, ** denotes *p* < 0.01, *** denotes *p* < 0.001.

Given the potent ability of CO depot to remodel the inflammatory microenvironment, we further explored whether it could promote high‐quality, scarless tissue healing following infection. Chronic infected wounds after 15 days of treatment were subjected to comprehensive histological and immunofluorescence staining to analyze the underlying repair mechanisms. H&E staining revealed that compared with the PBS group, the S3fC gel+US group showed a significant reduction in inflammatory infiltration (immune cell count) (Figure 9a and Figure S33). Moreover, H&E and Masson staining demonstrated that S3fC gel combined with US treatment enhanced collagen deposition and granulation tissue thickness at the infection site, further promoting re‑epithelialization and fibroblast proliferation (Figure 9a). The blue areas indicated abundant mature collagen arranged in a basketweave pattern. These findings support the concept that S3fC gel+US reduces acellular debris and neutrophil infiltration while enhancing collagen deposition and remodeling, thereby driving infected tissue into the proliferative phase of repair.

Ki67, a widely recognized nuclear proliferation marker, was employed to assess the proliferation of fibroblasts and keratinocytes [60]. The S3fC gel+US group was more competent than the other groups in advancing the expression of Ki67‐positive cells (Figure 9b,d), which indicated that the S3fC gel under US irradiation could facilitate the proliferation of cells by the biological activity of anti‐inflammation. CD31, also known as platelet endothelial cell adhesion molecule‐1 (PECAM‐1), drives angiogenesis by mediating endothelial cell adhesion and modulating vascular permeability. We examined CD31 expression in infected tissues across five treatment groups and found the highest level in the S3fC gel+US group, where clear vascular outlines were observable—a critical factor for post‐infection repair (Figure 9b,e). In addition, yes‐associated protein (YAP) is responsible for regulating hair follicle stem cell function and the telogen‐to‐anagen transition in the hair growth cycle [61]. Studies indicate that local targeted inhibition of YAP is a promising strategy to promote scarless healing and hair follicle regeneration [62]. Compared to the control group, YAP expression was significantly downregulated after S3fC gel+US treatment, with a lesser reduction following SC gel+US, suggesting that the CO depot can steer the healing trajectory toward scarless regeneration by targeting YAP (Figure 9b,f). Under persistent inflammatory or injurious stimulation, the repair process becomes dysregulated, leading to excessive and disordered deposition of type I collagen and a relative deficiency of type III collagen. We subsequently analyzed collagen subtypes during post‐infection repair. Sirius red staining revealed that the S3fC gel+US group exhibited the lowest ratio of type I to type III collagen at day 15, indicating a gradual restoration of type III collagen during the repair process (Figure 9c,g). Successful repair of infected tissue depends on the spatiotemporal synergy and dynamic balance between type I and type III collagen.

As previously established, vascular is essential for tissue repair, supplying oxygen, nutrients, and growth factors. Inadequate or dysfunctional angiogenesis often prevents healing or leads to fibrosis in chronically infected tissues. To further assess in vivo repair outcomes post‐infection, 3D confocal microscopy fluorescence imaging with near‐infrared probes was employed to visualize vascular distribution. SD rats treated with the CO depot were injected with Alexa Fluor 647‐conjugated anti‐CD31 antibody, revealing well‐established vasculature in the infected tissue (Figure 9h). In contrast, vasculature in the PBS‐treated control group remained in a nascent stage, re‐emphasizing the critical role of CO in promoting neovascularization. The 3D reconstruction vividly depicts the distribution of new blood vessels within the regenerating tissue. Subsequent analysis of α‐SMA coverage was used to assess vascular maturity, which reflects the extent of pericyte and smooth muscle cell support for endothelial tubes [63]. 3D imaging using cyanine dye FD‐1080‐conjugated anti‐α‐SMA antibody demonstrated significantly higher vascular maturity in CO depot‐treated samples, indicating entry into the remodeling phase with stabilized vasculature (Figure 9i). This may be attributed to the sustained release of CO coupled with the growth factor‐enriched SIS, which together orchestrates a comprehensive pro‐regenerative program. Crucially, the sustained, localized release from the CO depot is pivotal, as it directly inhibits the inflammatory NF‐κB/MAPK/IRF3 pathways and induces a reparative macrophage polarization. This anti‐inflammatory shift fosters a conducive microenvironment in which the SIS collagen provides trophic support for angiogenesis, thereby effectively promoting neovascularization. Consequently, this collaborative cascade accelerates the regeneration of key structures, including hair follicles and the favorable type III collagen, steering the healing process toward functional tissue restoration rather than mere scarring (Figure 9j).

### 2.10 In Vivo Fate and Safety Assessment

Having demonstrated the therapeutic efficacy of the CO depot in both acute and chronic infection models, we next evaluated its systemic safety and in vivo fate. To assess CO‑related systemic exposure, carboxyhemoglobin (COHb) levels were measured in rat blood at 0, 1, 5, 10, and 15 days after S3fC gel implantation plus US activation. COHb levels remained below 5.0% at all time points (Figure S34a), far below the clinical toxicity threshold of 10–15% for mild symptoms [64]. Serum HO‑1 levels, an indicator of cellular CO exposure, were moderately elevated in the S3fC gel+US group during the first 5 days, increasing by approximately 6.4% compared with the control group, and returned to baseline by day 15 (Figure S34b), indicating a controlled and reversible systemic response. In addition to these systemic markers, we directly quantified local CO concentrations in wound tissue using a CO‑sensitive electrode calibrated against GC (Figure S31). The local tissue CO concentration remained below 5.5 µM over 48 h. This value is an order of magnitude lower than the inhaled CO concentration (100–250 ppm) typically required to achieve anti‑inflammatory effects or to elicit measurable toxicity in rodent studies [65]. Moreover, it is well below the estimated local toxic threshold (∼50 µM) derived from the literature [66]. Therefore, our system maintains local CO levels within a safe and effective therapeutic window while avoiding systemic toxicity.

Beyond CO‑related safety, we also investigated the long‑term fate of the COFQD frameworks. A 96‑h pharmacokinetic study was conducted after intravenous injection of 3HF@COFQDs. The blood concentration‑time curve over 96 h indicated that Mn levels peaked within the first few hours and then declined progressively, with little remaining by 96 h (Figure S35a). Organ distribution analysis over 48 h showed that Mn initially accumulated in the liver and kidney within 10 h, followed by a gradual decrease; by 48 h, Mn levels in all six organs (heart, liver, spleen, lung, kidney, and brain) had dropped to near‑baseline levels (Figure S35b,c). At 15 days post‑injection, Mn levels in the same six organs were measured; no significant Mn accumulation was observed in any examined organ, with only about 3.1% of the initial Mn remaining in the liver (Figure S36a). Mn excretion was tracked in urine and feces at 0.5, 1, 5, 10, and 15 days. ICP‑MS results showed that Mn was cleared mainly through urine and feces, with most eliminated by 15 days (Figure S36b). H&E staining of major organs revealed no obvious damage or inflammation (Figure S37). Complete blood count (WBC, RBC, etc.), serum biochemical indices (ALT, AST, and BUN), electrolyte (Ca^2+^, K^+^, and Cl^−^), and blood gas parameters (pH, pCO_2_, and pO_2_) were also measured at day 15. All values fell within normal ranges and showed no significant difference from the healthy control group (Figures S38 and S39).

Regarding the COF skeleton itself, its potential degradation products were considered. The boronate ester bonds in the COFQDs are hydrolytically labile under mild acidic or enzymatic conditions; therefore, the main degradation products are likely low‑molecular‑weight boron‑containing oligomers and porphyrin derivatives. Previous reports have shown that similar molecules can be cleared via renal and hepatic pathways [67]. Moreover, due to the ultrasmall size (∼5 nm) of the COFQDs, their hydrolytic degradation efficiency is expected to be much higher than that of 2D and 3D COF materials, eventually leading to clearance via hepatic and renal filtration [68]. Overall, these data demonstrate that the COFQDs and their degradation products do not cause long‑term accumulation or organ toxicity. The ultrasound frequency applied in this study (1.0 MHz) lies within the clinically relevant range for therapeutic ultrasound and enables efficient acoustic activation of gas release without inducing significant thermal effects. The brief, non‐invasive irradiation protocol further supports the feasibility of this approach for wound care applications. Collectively, these optimized acoustic parameters offer a favorable clinical safety window and guarantee high patient compliance due to the painless nature of the treatment, thereby reinforcing the clinical translational potential of this therapeutic strategy.

## 3 Conclusions

In summary, we have proposed and validated a novel gasotransmitter delivery and action strategy based on an ultrasound‐responsive CO depot. Compared with conventional systemic or untargeted CO delivery, the “rigid storage–flexible release” depot designed with Mn‐immobilized COF quantum dots embedded in a decellularized ECM gel possesses several key advantages: i) The rigid porous structure of COFs, with Mn sites forming σ‐π bonds, provides precise, high‐capacity storage for CO gas molecules, preventing premature leakage; ii) The flexible ECM polymer network acts as a smart gate, slowly degrading to sustainably release CO for up to 10 days, thereby minimizing systemic toxicity; iii) The released CO effectively suppresses the NF‐κB/MAPK/IRF3 signaling pathways, reprograms the inflammatory microenvironment; iv) This system achieves precision therapy through a time‐programmatically controlled treatment regimen integrating antimicrobial therapy, anti‐inflammatory intervention, and tissue remodeling. In MRSA‐infected models of acute and chronic wounds, this CO depot not only effectively eradicated bacteria and accelerated wound closure but also significantly attenuated tissue inflammation and enhanced collagen deposition and angiogenesis. The depot exhibited excellent biocompatibility, causing no local or systemic toxicity, both in vitro and in vivo. Overall, our findings demonstrate an integrated platform for acoustically‐triggered long‐term CO delivery, which achieves sequential anti‐infection and post‐infection repair by integrating a rigid skeleton with a flexible network. This strategy provides a promising and translatable approach for treating MDR bacterial infections and may inspire the design of next‐generation gasotransmitter‐based therapeutics for managing infectious and inflammatory diseases.

## 4 Materials and Methods

### 4.1 Materials

2,3,6,7,10,11‐Hexahydroxytriphenylene hydrate (97%), mesitylene (98%, Extra Dry), acetonitrile (99%), methanol (analytical reagent grade), chloroform (analytical reagent grade), manganese(II) acetate tetrahydrate (99%), HNO_3_ (AR), and 2',7'‐dichlorodihydrofluorescein diacetate (97%) were purchased from Shanghai Saien Chemical Technology Co., Ltd. 5,15‐bis(4‐boronophenyl) porphyrin was obtained from our previous synthesis. 3‐Hydroxyflavone (95%), 1,3‐diphenylisobenzofuran (95%), 3,7‐bis(dimethylamino)phenothiazin‐5‐ium chloride (methylene blue, 95%), and lipopolysaccharides (>500 000 EU/mg) were obtained from Shanghai Bide Pharmaceutical Technology Co., Ltd. N‐Phenylnaphthalen‐1‐amine (99.98%), CO probe 1 (98.3%), and calcein acetoxymethyl ester (99.73%) were sourced from MedChemExpress LLC. All bacteriological culture media were purchased from Beijing Solarbio Science & Technology Co., Ltd. All experiments used ultrapure water (18.2 MΩ·cm at 25 ± 2°C) produced by a Milli‐Q Advantage A10 system (Millipore, Burlington, MA, USA).

### 4.2 Animals and Infected Models

Animal experiments were performed in accordance with protocols approved by the Animal Ethics Committee of Bengbu Medical University and complied with the guidelines of the Animal Care and Use Committee (Approval No. 2024528). Female BALB/c mice (7–8 weeks old, RRID:IMSR_CRL:028) were purchased from Nanjing Qinglongshan Animal Breeding Farm. First, mice were randomly divided into five groups (n = 5 per group) using the RAND function in Excel: (I) PBS, (II) SC gel, (III) S3fC gel, (IV) SC gel+US, and (V) S3fC gel+US. A full‐thickness circular wound (∼10 mm in diameter) was created on the dorsal skin of each mouse. The wound was then inoculated with 100 µL of MRSA (∼1×10^8^ CFU/mL) and covered with a bandage to promote infection, establishing an acute infection model. SC gel or S3fC gel (equivalent to 10 mg/kg of 3HF@COFQDs) was topically applied to the wound site. Mice in groups IV and V received ultrasound irradiation (1.2 W/cm^2^) for 20 min. Wound conditions were photographed and recorded with a digital camera. Animals were anesthetized with isoflurane during procedures and euthanized by cervical dislocation at the endpoint. Bacterial burden in the peri‐wound tissue was assessed by plate counting. On day 11, skin tissues from each group were fixed in 4% paraformaldehyde for histological analysis, including H&E staining, Masson's trichrome staining, and immunofluorescence staining. Sections were permeabilized with 0.1% Triton X‐100 for 30 min and blocked with commercial blocking buffer for 1 h. They were then incubated with primary antibodies against MPO (Abcam ab208670, RRID:AB_2864724), IL‑6 (Abcam ab259341, RRID:AB_2927381), and TNF‑α (Abcam ab183218, RRID:AB_2889388), followed by appropriate secondary antibodies. Nuclei were counterstained with DAPI. Stained sections were examined using CLSM and analyzed with ImageJ software.

For the chronic infection model, male Sprague‑Dawley rats (∼12 weeks old, RRID:RGD_734476) were obtained from the same breeding farm. After one week of acclimatization, diabetes was induced by a single intraperitoneal injection of freshly prepared streptozotocin (STZ, 60 mg/kg in 1% citrate buffer) following a 12‑h fast. Control rats received an equal volume of sterile citrate buffer. Random blood glucose was measured 72 h post‑injection via tail‑vein sampling; rats with glucose levels ≥16.7 mmol/L were considered diabetic and stabilized for 5 days before wounding. A full‑thickness circular skin wound (∼15 mm in diameter) was created on the dorsal surface and inoculated with 50 µL of MRSA suspension (∼1×10^8^ CFU/mL) to induce secondary infection. The administration regimen for the five groups was identical to that in the acute infection model but administered at twice the dosage. On day 15, a subset of rats was euthanized, and MRSA‑infected skin (along with adjacent muscle) was collected. Soft tissues were digested in cold sterile PBS, filtered through a 40 µm cell strainer, and the resulting cell suspension was stained with antibodies against F4/80 (Abcam ab111101, RRID:AB_10859466), CD86 (Abcam ab239075, RRID:AB_2927417), and CD206 (Abcam ab64693, RRID:AB_1523910) for flow cytometric analysis of macrophage polarization. Residual bacteria in peri‑wound tissues were quantified by plate counting. Skin tissues were fixed in 4% paraformaldehyde for H&E staining, Masson's trichrome staining, and Sirius red staining. Immunofluorescence was performed to detect F4/80 (Abcam ab111101), CD86 (Abcam ab239075), CD206 (Abcam ab64693), iNOS (Abclonal A3774, RRID:AB_3094627), active YAP1 (Abcam ab205270, RRID:AB_2813833), CD31 (Abcam ab182981, RRID:AB_2920881), and Ki67 (Proteintech 27309‐1‐AP, RRID:AB_2756525). To evaluate in vivo biosafety, major organs, including heart, spleen, lung, liver, and kidney, were harvested for further examination. Serum biochemical indices were measured using commercial colorimetric assay kits (Thermo Fisher Scientific) following the manufacturer's protocols. Electrolyte concentrations were determined by the ion‑selective electrode method (EasyLyte Plus). Blood gas parameters were analyzed using a blood gas analyzer (Premier3500) with disposable sensor cartridges. All measurements were performed on serum samples collected at the indicated time points. Animals were humanely euthanized at the end of the study.

### 4.3 Statistical Analysis

Unless otherwise specified, in vitro experiments were independently repeated three times, and sample sizes for in vivo experiments are indicated in the corresponding figure legends. Quantitative data are presented as mean ± standard deviation (SD), with the specific sample size (n) for each experiment detailed in the corresponding figure legends. Statistical significance was assessed using two‐sided Student's *t*‐test, as appropriate, in GraphPad Prism 10. A *p*‐value of less than 0.05 was considered statistically significant, denoted as *p < 0.05, **p < 0.01, and ***p < 0.001.

## Author Contributions

B.S. conceived the idea and designed the project. B.S., F.H., C.Z., Z.Y., J.M., J.C., J.Z., Z.B., T.W., H.W., and X.Z. performed the experiments and analyzed the results. B.S., T.M., and Y.S. assisted with the figure production and experiment design. B.S. wrote and revised the original draft of the manuscript. B.S., Y.L., and Y.S edited the manuscript. B.S., Y.L., and Y.S supervised the whole project. All authors discussed the results and commented on the manuscript.

## Conflicts of Interest

The authors declare no conflicts of interest.

## Supporting information




**Supporting File**: advs76681‐sup‐0001‐SuppMat.docx.

## Data Availability

The data that support the findings of this study are available on request from the corresponding author. The data are not publicly available due to privacy or ethical restrictions.
