# Supplementary material for: Harmonizing Rigidity and Flexibility: Embedding COF Quantum Dots Into Extracellular Matrix Gel as Carbon Monoxide Depot for Acoustically Triggered Time‐Programmable Anti‐Infective Therapy
Source: Adv Sci (Weinh). 2026 Jul 20;13(57):e76681. doi: 10.1002/advs.76681 (PMC13383690; doi:10.1002/advs.76681)
Supplement: Supplementary file 1 [file ADVS-13-e76681-s001.docx]

*Supporting Information for*

**Harmonizing Rigidity and Flexibility: Embedding COF Quantum Dots into Extracellular Matrix Gel as Carbon Monoxide Depot for Acoustically Triggered** **Time-Programmable Anti-Infective Therapy**

*Baohong Sun^1^*, Fang Han^1^, Chunxiao Zhu^1^, Zhiyuan Yang^2^, Haoru Wang^3^, Jinpei Mei^1^, Jie Chen^1^, Jingwen Zhu^1^, Zihan Bo^1^, Taju Wu^4^, Xiaogang Zhou^5^, Tao Ma^1^*, Yutian Su^3^*, Youhui Lin^2^**

^1^ Interdisciplinary Eye Research Institute (EYE-X Institute), Anhui Engineering Technology Research Center of Biochemical Pharmaceutical, School of Pharmacy, Bengbu Medical University, Bengbu 233030, P. R. China

E-mail: baohongsun7@bbmu.edu.cn (B. Sun), matao@bbmu.edu.cn (T. Ma)

^2^ Department of Physics, Research Institute for Biomimetics and Soft Matter, Fujian Provincial Key Laboratory for Soft Functional Materials Research, Xiamen University, Xiamen 361005, P. R. China

E-mail: linyouhui@xmu.edu.cn (Y. Lin)

^3^ State Key Laboratory of Respiratory Disease, National Clinical Research Center for Respiratory Disease, Guangzhou Institute of Respiratory Health, The First Affiliated Hospital of Guangzhou Medical University, National Center for Respiratory Medicine, Guangzhou 510120, P. R. China

E-mail: 2023390241@gzhmu.edu.cn (Y. Su)

^4^ School of Life Science, Bengbu Medical University, Bengbu 233030, P. R. China

^5^ Anhui Key Laboratory of Infection and Immunity, School of Basic Medicine, Bengbu Medical University, Bengbu 233030, P. R. China

**1. Materials and characterizations**

**1.1 Characterizations**

The surface composition and elemental distribution of the samples were analyzed using a transmission electron microscope (TEM, Tecnai G2 F30 S-TWIN) operated at an accelerating voltage of 200 kV. The surface morphology was observed by scanning electron microscopy (SEM, ZEISS Sigma 300, Germany). The height profiles were measured with an atomic force microscope (AFM, Bruker Dimension ICON). X-ray photoelectron spectroscopy (XPS) was performed on a PHI5000 VersaProbe spectrometer (ULVAC-PHI, Japan). The crystal structure was examined by powder X-ray diffraction (XRD, Shimadzu Evolution, Japan). The mesoporous structure was characterized via nitrogen adsorption-desorption measurements using an automated surface area and porosity analyzer (BET, Micromeritics ASAP 2460). Inductively coupled plasma optical emission spectrometry (ICP-OES, PerkinElmer AVIO200) and inductively coupled plasma mass spectrometry (ICP-MS, Agilent 7800) were used to quantify manganese (Mn) content. The chemical structure was analyzed by cross-polarization magic angle spinning solid-state nuclear magnetic resonance spectroscopy (CP/MAS NMR, Bruker Avance Neo 400WB, Germany). Ultraviolet visible (UV-Vis) absorption spectra were recorded on a Shimadzu UV-3600 spectrophotometer. Chemical bond information was obtained by Fourier-transform infrared spectroscopy (FT-IR, Nicolet iS 10 series). In situ diffuse reflectance infrared Fourier transform spectroscopy (DRIFTS) was performed on a Thermo Scientific Nicolet iS50 FTIR spectrometer equipped with an in situ diffuse reflectance cell (Harrick, Praying Mantis). High-resolution mass spectrometry (HRMS) was conducted on an SCIEX X500B QTOF mass spectrometer. Electron spin resonance (ESR) measurements were acquired on a Bruker EMXplus spectrometer. The particle size distribution and surface charge were determined by dynamic light scattering (DLS, Malvern). Cellular fluorescence images were captured using a Nikon A1R confocal laser scanning microscope (Nikon, RRID:SCR_020317). Western blotting results were visualized with a high-sensitivity chemiluminescence imaging system (Bio-Rad ChemDoc, Singapore). Flow cytometry analysis was performed on a BD Accuri C6 Plus flow cytometer. *In vivo* observations were carried out using a small animal real-time imaging system (PE IVIS Lumina XR III, PerkinElmer, USA).

**1.2 Synthesis of COFs**

Synthesis of 5,15-bis(4-boronophenyl)-porphyrin manganese(II): The synthesis of 5,15-bis(4-(1,3,2-dioxaborinan-2-yl)-phenyl)porphyrin was performed according to our previous literature ^[1]^. Under an argon atmosphere, the reactant [5,15-bis(4-(1,3,2-dioxaborinan-2-yl)-phenyl)porphyrin] (100 mg) and an excess of Mn(OAc)_2_·4H_2_O (100 mg) were dissolved in 25 mL of DMF. The mixture was gently refluxed at 90-110°C in the dark for 4-6 hours. After completion of the reaction, the mixture was cooled to room temperature and slowly poured into an ice-water mixture to precipitate the product. The precipitate was collected by filtration and washed with water and methanol. The product was purified by column chromatography using DCM/methanol (DCM: methanol = 97:3). The obtained product was then stirred in a mixture of THF (450 mL) and aq. HCl solution (450 mL, pH = 1) at room temperature in the dark. After 24 hours, the reaction was quenched by adding 650 mL of CH_2_Cl_2_. The solid product was obtained by filtration (62% yield), washed five times with methanol, and dried under vacuum. Mass spectrum: Calculated 609.16; Experimental: 609.8525.

Porphyrin-based COFs were synthesized via a solvothermal method. Specifically, 12.2 mg of 5,15-bis(4-boronophenyl)-porphyrin manganese(II) (Por, 0.02 mmol) and 4.5 mg of 2,3,6,7,10,11-triphenylenehexol (HHTP, 0.014 mmol) were added to 1 mL of a mixed solvent (acetonitrile/mesitylene = 7:3, v/v) in a sealed, heat-resistant Pyrex tube. The suspension was sonicated for 10 min to fully disperse the monomers. The Pyrex tube was then degassed through three freeze-pump-thaw cycles and heated at 120°C for 72 h. After cooling the mixture to room temperature, the suspension was filtered through a Hirsch funnel, and the precipitate was collected. The precipitate was washed five times with toluene and ethanol, and isolated from the suspension via centrifugation at 10,000 rpm for 10 min. Finally, the precipitate was further dried under dynamic vacuum at 120°C for 12 h to yield the activated COFs.

**1.3 Synthesis of COFQDs**

A low-temperature-mediated ultrasonic exfoliation and self-hydrolysis approach was employed to cleave the boronate ester linkages in bulk layered COFs. The procedure involved the following steps: First, a low-temperature pretreatment was conducted by submerging the powdered starting material of two-dimensional COFs in liquid nitrogen for 30 minutes. Subsequently, the frozen powder was immediately dispersed into a mixed solvent of isopropanol and water (pH 4.5) with a volume ratio of 1:1. Liquid-phase exfoliation was then carried out with the assistance of an ultrasonic bath. The initial concentration of the 2D COF material in the dispersion was 1 mg/mL. Following the low-temperature-mediated exfoliation and cleavage treatment, the resulting dispersion was centrifuged at 5000 rpm for 15 minutes to remove unexfoliated material. To further isolate the COF quantum dots (COFQDs) from the mixture, the supernatant was collected using a pipette and subjected to vacuum filtration through an ultrafine membrane filter (Millipore) with a pore size of 25 nm. The synthesis yield was calculated to be 20.8% using the following formula: Synthesis yield = (dry mass of COFQDs/total dry mass of COFs) × 100%. It should be noted that the obtained concentration of quantum dots is likely an underestimate, as many are presumably deposited on the remaining nanosheets and removed during filtration.

**1.4 Preparation of 3-HF@COFQDs**

One hundred milligrams of COF quantum dots (COFQDs) were dispersed in 20 mL of deionized water. Following this, a solution of 3-hydroxyflavone (3-HF, 3 mg/mL in ethanol/water) was added to the aforementioned dispersion. The mixture was then subjected to vigorous stirring for 12 hours and subsequent ultrasonication for 2 hours. After stirring, the mixture was centrifuged at 13,000 rpm for 30 minutes. The solid precipitate was separated, and the supernatant was collected. The collected supernatant was dialyzed against deionized water using a membrane with a molecular weight cut-off (MWCO) of 500-1000 Da for 36 hours. Finally, the product was obtained by freeze-drying (lyophilization).

**1.5 Preparation of SIS gel**

The porcine small intestinal submucosa (SIS) was decellularized through a standardized and optimized protocol involving mechanical separation, delipidation, enzymatic digestion, detergent treatment, freeze-drying, and sterilization ^[2]^. Briefly, within several hours post-slaughter, the entire small intestine was harvested from inspected healthy pigs. It was thoroughly rinsed with ice-cold sterile saline, placed on ice, and cut into approximately 12 cm long segments. The serosa and muscular layers were then completely removed via meticulous mechanical scraping to obtain the initial SIS material—a semi-transparent white membrane consisting primarily of the collagenous submucosa. A systematic series of chemical and biological treatments is followed to thoroughly remove cellular components. The SIS membranes were immersed in a 1:1 (v/v) mixture of methanol and chloroform under a fume hood for 12 hours to dissolve and remove lipids. This was followed by extensive rinsing with deionized water to ensure complete removal of residual organic solvents. Subsequently, the membranes were transferred into a solution containing 0.25% trypsin and 0.02% EDTA and incubated at 37°C for 12 hours to enzymatically digest residual proteins and cellular debris within the extracellular matrix. Next, to remove nucleic acids and remaining proteins, the samples were incubated in a solution of 1 mol/L sodium chloride and 0.1% sodium dodecyl sulfate (SDS) with constant agitation for 4 hours. Finally, the samples were treated with a 0.1% peracetic acid/ethanol solution (1:9, v/v) for 30 minutes for effective sterilization and further oxidative removal of antigens. After each chemical step, the samples were thoroughly rinsed with sterile saline. Following these treatments, the purified SIS samples were freeze-dried to form porous scaffolds. Terminal sterilization was then performed by exposure to 254 nm ultraviolet light for 60 minutes, completing the preparation of the base SIS scaffolds. For hydrogel formation, the SIS scaffolds were fully hydrated by immersion in deionized water at 4°C for 12 hours to obtain the SIS gel.

**1.6 Fabrication of SC gel and S3fS gel**

Two dried, porous, and sterilized SIS scaffolds (~2 cm×2 cm, thickness ~0.5 mm), prepared via the aforementioned protocol, were subjected to differential functional loading to construct the experimental and control materials. Briefly, one scaffold, serving as the loading control, was immersed in 20 mL of an aqueous dispersion of pure COFQDs at a concentration of 0.5 mg/mL. The other scaffold, serving as the drug-composite experimental group, was immersed in an equal volume (20 mL) of an aqueous dispersion containing 3-hydroxyflavone-loaded COF quantum dots (3-HF@COFQDs) at an identical QD mass concentration of 0.5 mg/mL. Both samples were incubated statically at a constant temperature of 4°C under light-protected conditions for 24 hours. This process facilitated the sufficient penetration of nanoparticles into the porous three-dimensional network of the scaffolds via diffusion and their stable loading through physical adsorption. After incubation, the scaffolds were carefully retrieved using sterile forceps. Excess surface liquid was gently drained, and the scaffolds were placed on pre-cooled lyophilization trays. Subsequently, they were transferred together to a freeze-dryer. They were first pre-frozen at -50°C for 4 hours, followed by primary drying under a vacuum of < 10 Pa for 24 hours to completely remove residual moisture, yielding the loaded dry composite scaffolds. Finally, prior to use, each type of freeze-dried scaffold was individually immersed in an adequate volume of sterile phosphate-buffered saline (PBS, pH 7.4) and rehydrated at 4°C for 2 hours to restore a soft, moist hydrogel state. This resulted in the preparation of two distinct hydrogels: the SIS composite hydrogel loaded solely with COFQDs, designated as the SC gel, and the drug-functionalized composite hydrogel loaded with 3-HF@COFQDs, designated as the S3fC gel. Both gels were stored at 4°C for subsequent physicochemical characterization and biological experiments.

**1.7 ESR measurement**

Singlet oxygen (1O2) generated from 3-HF@COFQDs upon ultrasonic irradiation was evaluated using 2,2,6,6-tetramethyl-4-piperidone (TEMP) as a spin-trapping agent. Specifically, 50 µL of a 3-HF@COFQDs dispersion (2.5 mg/mL) was mixed with 50 µL of an aqueous TEMP solution (500 mM) in a sealed vial. The mixture was then subjected to ultrasonic irradiation for 60 seconds using an ultrasonic device operating at a frequency of 1 MHz and an intensity of 1.8 W/cm^2^. Immediately after sonication, approximately 40 µL of the solution was transferred into an electron spin resonance (ESR) tube using a quartz capillary tube. Finally, the ESR signal was measured at room temperature with a Bruker EMXplus spectrometer.

**1.8 SDT measurement**

The sonodynamic therapy (SDT) effect of 3-HF@COFQDs was evaluated using the 1,3-diphenylisobenzofuran (DPBF) assay and the methylene blue (MB) method. For the DPBF/MB assay, a solution of 3-HF@COFQDs (different concentrations) was added to DPBF (40 µg/mL), followed by ultrasound (US) treatment. The UV-Vis absorption spectra of the samples were measured at 420 nm and 665 nm using a UV-Vis spectrophotometer. The duration of ultrasound treatment ranged from 0 to 5 minutes. For the electrochemical measurement, ultrasonic current was monitored using an electrochemical workstation. A working electrode was prepared by depositing a suspension of 3-HF@COFQDs (200 µL) onto an indium tin oxide (ITO)-coated glass slide in a Na_2_SO_4_ electrolyte solution (0.2 mol/L). The transient current changes of the 3-HF@COFQDs under ultrasonic irradiation (3.0 MHz, 3 W/cm^2^) were recorded.

**1.9 CO storage and releasing**

The CO storage and release were monitored using a commercially available CO detector (Dräger Pac). Briefly, measurements were conducted in a sealed desiccator equipped with the detector. A scintillation vial containing 3-HF@COFQDs dispersed in deionized water or PBS buffer (200 μg/mL) was placed inside the desiccator along with a magnetic stir bar. After sealing the lid, the dispersion was treated with US at intensities of 0.6, 1.2, and 1.8 W/cm^2^ for 20 minutes. The CO detector measured the concentration of CO in the gas phase, with data recorded at regular intervals. The amount of released CO was calculated according to the following formula ^[3]^:

N_CO_ = *pV_g_*/*RT* + *cV_l_* =*p* (*V_g_/RT* + *V_l_/k*)

where p is the partial pressure of CO; V_g_ and V_l_ are the volumes of the gas and liquid phases, respectively; R is the universal gas constant; T is the temperature; c is the concentration of CO in the liquid phase; and k is Henry's law constant for CO in water.

**1.10 Spread plate method**

Gram-negative *Escherichia coli* (ATCC 25922, RRID:BAL_25922) and Gram-positive methicillin-resistant *Staphylococcus aureus* (MRSA, ATCC 43300, RRID:BAL_43300) were employed as model bacterial strains. Bacterial suspensions were treated with the following five regimens: (1) PBS (control); (2) COFQDs; (3) 3-HF@COFQDs; (4) COFQDs+US; (5) 3-HF@COFQDs+US. Specifically, each sample (at a concentration of 150 μg/mL) was introduced into a bacterial suspension with a density of 1×10^7^ CFU/mL. For the groups designated for ultrasound treatment, the suspensions were exposed to US irradiation at an intensity of 1.2 W/cm^2^ for 20 minutes. All groups were subsequently incubated at 37°C for 2 hours. Following incubation, 30 μL of each treated bacterial suspension was plated onto Luria-Bertani (LB) agar plates and cultured at 37°C for 24 hours. Finally, the colony-forming units (CFUs) were enumerated using the standard plate counting method. The corresponding antibacterial rate was calculated according to the following formula:

Antibacterial Rate (%) = (1−*N_c_* / *N_t_*) × 100

where N_t_ represents the number of CFUs in the untreated control group, and N_c_ represents the number of CFUs in the treated group.

Furthermore, the antibacterial efficacy of the samples at varying concentrations (8, 16, 32.5, and 75 μg/mL) under US irradiation (1.2 W/cm^2^) was also evaluated. After the respective treatments, 30 μL of each bacterial suspension was plated onto LB agar and incubated at 37°C for 24 hours. The colony counts were then determined via the plate counting method.

**1.11 Live/dead staining**

The viability of bacteria in different treatment groups was assessed using the SYTO-9/PI live/dead double-staining assay (Thermo Fisher Scientific, L7012). The treatment procedures for the bacterial samples were consistent with those described for the standard plate count method. Following treatment, bacterial suspensions from all groups were co-stained with propidium iodide (PI, a red fluorescent nucleic acid dye) and SYTO-9 (a green fluorescent nucleic acid dye) for approximately 30 minutes in the dark and subsequently washed with phosphate-buffered saline (PBS). Bacterial viability was then examined under a fluorescence microscope, where live bacteria with intact membranes exhibited green fluorescence, and dead bacteria with compromised membranes exhibited red fluorescence.

**1.12 Morphology observation of bacteria**

Bacterial cells of the two strains in the logarithmic growth phase (~1×10^7^ CFU/mL) were co-incubated with PBS, COFQDs, or 3-HF@COFQDs (150 µg/mL), with or without concurrent ultrasound (US) irradiation. Following incubation, the bacterial cells were collected by centrifugation at 6000 rpm for 5 minutes and washed three times with 0.1 M phosphate-buffered saline (PBS, pH 7.2-7.4). The washed cells were then fixed with 2.5% (v/v) glutaraldehyde in PBS at 4°C for 6 hours. Subsequently, the fixed cells were dehydrated through a graded ethanol series (25%, 50%, 75%, 90%, and 100%). The samples were mounted on SEM stubs, air-dried under vacuum, sputter-coated with a thin layer of gold, and finally observed using a SEM.

For TEM observation, *E. coli* and MRSA cells cultured in LB medium were washed three times with PBS buffer and resuspended in PBS at a density of 1×10^6^ CFU/mL. The suspensions were then co-incubated with the respective samples (150 µg/mL). After incubation, the bacterial cells were pre-fixed overnight with 2.5% glutaraldehyde solution at 4°C, followed by post-fixation with 1% osmium tetroxide (OsO_4_) for 2 hours at 4°C. The fixed cells were washed three times with PBS buffer and sequentially dehydrated using a graded ethanol series (50%, 75%, 90%, 95%, and 100% ethanol) and a mixture of ethanol and acetone (1:1, v/v). The dehydrated cells were infiltrated with a mixture of acetone and epoxy resin (1:2, v/v) for 12 hours, followed by complete immersion in pure epoxy resin for polymerization. Finally, the resin-embedded blocks were sectioned into ultrathin slices, which were placed on carbon-coated grids and observed under a TEM at a low accelerating voltage to examine the morphological alterations of the bacterial cells.

**1.13 Protein leak assay**

Bacterial suspensions with a concentration of 1×10^7^ CFU/mL were incubated with COFQDs or 3-HF@COFQDs (150 μg/mL), with or without subsequent US treatment. For the groups subjected to US, the samples were irradiated at an intensity of 1.2 W/cm^2^ for 20 minutes. After a one-hour incubation period, the bacterial suspensions were collected, and the supernatants were diluted accordingly. Finally, the release of proteins into the supernatant, as an indicator of membrane integrity disruption, was quantified by measuring the absorbance at 280 nm using an enzyme-linked immunosorbent assay (ELISA).

**1.14 Bacterial growth kinetics**

MRSA suspensions (1×10^7^ CFU/mL) were treated with PBS (control), COFQDs (150 μg/mL), or 3-HF@COFQDs (150 μg/mL), followed by either exposure to or omission of US irradiation. For the US-treated groups, the samples were irradiated at an intensity of 1.2 W/cm^2^ for 20 minutes. After treatment, the bacterial suspensions from the different groups were incubated at 37°C. Bacterial growth was then monitored by measuring the optical density at 600 nm (OD600) over a period of 300 minutes.

**1.15 ROS detection**

The intracellular reactive oxygen species (ROS) levels in MRSA were evaluated using the fluorescent probe 2',7'-dichlorodihydrofluorescein diacetate (DCFH-DA). MRSA suspensions were first incubated with 10 μmol/L DCFH-DA at 37°C for 20 minutes. The mixture was then centrifuged at 3000 rpm for 5 minutes, washed three times with PBS, and resuspended in PBS, COFQDs (150 µg/mL), or 3-HF@COFQDs (150 µg/mL) solutions, respectively. The bacterial suspensions were incubated at 37°C for 6 hours. During this period, the US-treated groups were exposed to US irradiation at 1.2 W/cm^2^ for 20 minutes, while the corresponding control groups were kept under dark conditions without US irradiation. Following incubation, the fluorescence intensity of intracellular ROS in MRSA cells was measured using BD FACSCalibur flow cytometry (BD Biosciences, RRID:SCR_013311), and quantitative analysis was performed with FlowJo software (FlowJo, RRID:SCR_008520).

**1.16 Assessment of bacterial membrane permeability**

The integrity of the plasma membrane was assessed using the fluorescent probe N-phenyl-1-naphthylamine (NPN; MedChemExpress, HY-W009756) ^[4]^. Mid-log phase MRSA cells were washed and resuspended in HEPES buffer (5 mmol/L HEPES, pH 7.4, containing 20 mmol/L glucose) to a density of 1×10^7^ CFU/mL. The bacterial suspension was then co-incubated with NPN and the nuclear dye Hoechst 33342 until a stable fluorescence baseline was achieved. Subsequently, 100 µL of the mixture was added to the wells of a microplate containing an equal volume of either PBS (control), COFQDs, or 3-HF@COFQDs (both at 150 µg/mL in PBS). The samples were then either left untreated or subjected to US irradiation (1.2 W/cm^2^ for 20 min). Membrane damage was visualized using a Nikon A1R confocal laser scanning microscope. Quantitative analysis of membrane damage over time was performed by measuring fluorescence intensity with a Synergy 2 microplate reader (BioTek Instruments, Winooski, VT, USA).

**1.17 Decellularized assessment of SIS**

To determine whether cellular components remained in the prepared SIS, quantitative PCR (qPCR) was performed to detect DNA of the immune receptor gene DAP12 ^[5]^. Briefly, fresh and decellularized SIS samples (approximately 1 cm^2^ each) were accurately weighed and digested in a buffer containing proteinase K (20 mg/mL) at 55 °C with rotation for 8 h. DNA was then purified by phenol‑chloroform extraction and ethanol precipitation and finally dissolved in TE buffer. Genomic DNA extracted from porcine blood was used as a standard to generate a quantification curve. qPCR was carried out using primers specific to the DAP12 gene. The first-round PCR was performed in a 30 μL reaction mixture containing external primers, template DNA, MgCl_2_, dNTPs, and Taq polymerase. The thermal profile consisted of an initial step at 94°C for 5 min, followed by 15 cycles of 94°C for 20 s, 50°C for 30 s, and 72°C for 30 s. A portion of the first‑round product was used as a template for the second‑round PCR, in which internal primers and a fluorescence probe were added. All reactions were run on a PerkinElmer 2400 thermocycler, with a no‑template control included in each run. The threshold cycle (Ct) values were obtained during the exponential phase of amplification and converted into DNA copy numbers based on the standard curve. Final results were expressed as copy number per milligram of sample.

**1.18 Growth factors assessment**

The levels of growth factors, including VEGF, b-FGF, TGF-β, and Arg-1, in SIS samples were quantified using ELISA kits (Wuhan Fine Biotech Co., Ltd., Wuhan, China). Briefly, 100 mg of the SIS sample was placed in an ice bucket, mixed with 2 mL of ice-cold PBS buffer, and homogenized using a tissue homogenizer for 30 s. The homogenate was kept on ice for 10 min, and the homogenization step was repeated three times. Subsequently, the sample was centrifuged at 2000 rpm for 10 min. The supernatant was transferred to a fresh centrifuge tube and stored at 4 °C for subsequent ELISA analysis. All procedures were performed according to the manufacturer’s instructions. Each sample was analyzed in triplicate. Absorbance was measured at a single wavelength of 450 nm using a Synergy 2 microplate reader.

**1.19 Detection of intracellular CO concentration**

The release of CO was detected using a CO-specific fluorescent probe. MRSA or *E. coli* cells in the logarithmic growth phase were diluted with PBS to a concentration of 1×10^6^ CFU/mL. The bacterial cells were then treated with PBS (pH 7.2, control), SC gel, S3fC gel (1×1 cm pieces), or a solution of CO probe 1 (5 μM) combined with PdCl_2_ (5 μM). After incubation at 37°C for 30 minutes, the cells were collected, washed twice with PBS to remove any extracellular gel debris and probes, and resuspended in fresh PBS. The suspensions were subsequently either subjected to or withheld from US treatment (1.2 W/cm^2^, 20 minutes). Following irradiation, the cells were further incubated at 37°C for 30 minutes. Finally, cellular imaging was performed using a Nikon A1R confocal laser scanning microscope. For the green fluorescence channel, images were acquired with an excitation wavelength of 488 nm and an emission collection window of 500-580 nm.

**1.20 *In vitro* anti-inflammatory properties**

This study evaluated the protective effects of SC gel and S3fC gel against LPS-induced oxidative damage. The pre-treatment procedure was consistent with the protocol for intracellular ROS measurement. Inflammatory human umbilical vein endothelial cells (HUVECs; Chinese Academy of Sciences Cell Bank, Shanghai, China), induced by LPS, were co-incubated with SC gel or S3fC gel (1×1 cm) for 1.5 hours. During this incubation period, the cells were exposed to ultrasound (US) irradiation at an intensity of 1.2 W/cm^2^ for 20 minutes. Subsequently, intracellular ROS generation and distribution were detected using the fluorescent probe DCFH-DA at a concentration of 4.5 µM. Finally, a Nikon A1R confocal laser scanning microscope was employed to assess the inhibitory effects of SC gel and S3fC gel on oxidative stress.

**1.21 Macrophage polarization**

RAW 264.7 cells at 80% confluency were seeded in flasks. To establish the positive control for M1 polarization, cells were stimulated with 100 ng/mL lipopolysaccharide (LPS) for 24 hours. Untreated cells served as the negative control. M1-polarized macrophages were then treated for 12 hours with the following: SC gel (without US), S3fC gel (without US), SC gel combined with ultrasound (US, 1.2 W/cm^2^, 20 min), or S3fC gel combined with US. Following treatment, the cells were fixed, permeabilized, and blocked with bovine serum albumin (BSA) to prevent nonspecific binding. Subsequently, the cells were stained for 1 hour with the following primary antibodies: F4/80 (Abcam), CD86 (BD Biosciences, 553768, RRID:AB_395038), and CD206 (BD Biosciences, 565250, RRID:AB_2739133). The expression levels of the surface markers CD86 and CD206 were analyzed using a FACSCalibur flow cytometer (BD Biosciences). Cellular staining and morphology were further observed under a Nikon A1R confocal laser scanning microscope.

**1.22 Cytotoxicity and hemolytic assay**

All cell lines were obtained from Jiangsu KeyGEN BioTECH Corp., Ltd. (Nanjing, China) and maintained at 37°C with 5% CO2 in high-glucose DMEM supplemented with 10% FBS and 1% penicillin-streptomycin.

Cytotoxicity against RAW 264.7 cells was evaluated by MTT assay. Cells were seeded in 96-well plates (1×10^4^ cells/well) and treated for 24 h with varying concentrations (50, 100, 200 μg/mL) of COFs, COFQDs, 3-HF@COFQDs, or gel samples (SIS, SC, S3fC; 1×1 cm). PBS-treated cells served as the control. After treatment, 20 μL of MTT solution (5 mg/mL in PBS) was added per well and incubated for 4 h. Formazan crystals were dissolved with 100 μL DMSO, and absorbance was measured at 570 nm using a Synergy 2 microplate reader (BioTek Instruments, Winooski, VT, USA). Cell viability was calculated as:

Cell Viability (%) *= (OD_t_ / OD_c_)* × 100

where OD_t_ and OD_c_ are the absorbance of treated and control wells, respectively. Experiments were performed in triplicate.

For hemolysis testing, fresh RBCs were isolated from whole blood by centrifugation (3000 rpm, 6 min), washed, and resuspended in saline. Test samples in saline (1.2 mL) were mixed with RBC suspension (0.3 mL) and incubated at 37°C for 3.5 h. Saline and 1% Triton X-100 served as negative and positive controls, respectively. After centrifugation, supernatant absorbance at 570 nm was measured. Hemolysis percentage was calculated as:

Hemolysis (%) = *[(A_s_–A_n_)/(A_p_–A_n_)]*×100

where A_s_, A_n_, and A_p_ are the absorbance of the sample, negative control, and positive control, respectively.

**1.23 Transwell, proliferation, and cell migration assays**

For the Transwell migration assay, 100 μL of HUVEC cell suspension was seeded into the upper chamber of a Transwell insert (Corning). The lower chambers were filled with media containing the following treatment groups: PBS, SC gel, S3fC gel, SC gel+US (1.2 W/cm^2^, 20 min), and S3fC gel+US (1.2 W/cm^2^, 20 min). All treatments were prepared in DMEM supplemented with 1% FBS. After a 24-hour incubation, the cells that had migrated to the lower side of the membrane were fixed with 4% paraformaldehyde and stained with 0.1% crystal violet. The number of migrated cells was quantified by counting cells in five randomly selected fields per insert under a light microscope. For fluorescence-based proliferation observation, HUVECs were subjected to the same treatments as above for 24 hours. Subsequently, the cells were incubated with 6 μM Calcein AM probe (AAT Bioquest, 22002) for 20 minutes at 37°C to label viable cells. The fluorescent signal was then visualized and imaged using a Nikon A1R confocal laser scanning microscope. Finally, a scratch wound healing assay was performed to evaluate HUVEC migration. HUVECs were cultured until they reached approximately 85% confluence. A uniform scratch was created across the cell monolayer using a sterile pipette tip. The cells were then washed and treated with the same set of treatment groups (all in DMEM with 1% FBS). Cell migration into the scratch area was monitored and recorded at 24 and 48 h post-scratching to assess wound closure.

**1.24 Western blotting**

Total protein was extracted from RAW 264.7 cells treated with SC gel or S3fC gel, with or without US treatment, using RIPA lysis buffer supplemented with 2% phenylmethylsulfonyl fluoride (PMSF) and a phosphatase inhibitor cocktail. The protein concentration was determined using a BCA protein assay kit. Subsequently, 20 μg of protein samples were separated by sodium dodecyl sulfate-polyacrylamide gel electrophoresis (SDS-PAGE) at 80 V and then transferred onto polyvinylidene difluoride (PVDF) membranes. Following transfer, the membranes were blocked with rapid blocking buffer (Proteintech, Wuhan, China) at room temperature for 0.5 hours. They were then incubated overnight at 4°C with primary antibodies against IKKβ (Proteintech 15649-1-AP, RRID:AB_2122307), p-IκBα (Proteintech 82349-1-RR, RRID:AB_3073626), and p65 (Proteintech 80979-1-RR, RRID:AB_2918923). After washing, the PVDF membranes were incubated with the corresponding secondary antibodies at room temperature. Finally, protein signals were captured using a PE IVIS Lumina XR III chemiluminescent imager (PerkinElmer, Waltham, MA, USA) and quantified by analyzing the band intensities with ImageJ software (NIH, RRID:SCR_003070).

**1.25 RNA-seq analysis**

A transcriptomic study was conducted on RAW 264.7 cells subjected to different treatments. Briefly, the experiment comprised three groups: (1) Control group: untreated cells; (2) SC gel+US and S3fC gel+US groups: RAW 264.7 cells were cultured with SC gel and S3fC gel and simultaneously exposed to US irradiation (1.2 W/cm^2^) for 20 minutes within a 1-hour incubation period. Immediately after treatment, the cell samples were snap-frozen in liquid nitrogen. Total RNA was extracted from the cells using TRIzol reagent, followed by RNA sequencing. Differentially expressed genes (DEGs) were identified based on the criteria of |log2(fold change)| > 1 and a *P*-value < 0.05. The sequencing data were measured and analyzed by Nanjing Jisihuiyuan Biotechnology Co., LTD (Nanjing, China).

**1.26 qPCR analysis**

RNA extracted from three independent biological replicate samples was subjected to qPCR analysis. qPCR was performed using a CFX96 Multicolor Real-Time PCR Detection System (Bio-Rad, Hercules, CA, USA), and data analysis was managed via Bio-Rad CFX Manager software (RRID:SCR_026760). Amplification was carried out using a SYBR Green dye (KAPA SYBR® FAST qPCR Kit; Kapa Biosystems, Boston, MA, USA, strictly following the manufacturer's protocols. Each cDNA sample was analyzed in triplicate, and the mean Ct value was calculated. Gene expression levels were normalized to the endogenous Gapdh levels in RAW 264.7 cells. The relative expression levels were calculated using the 2*^−ΔΔCT^* method. Gene-specific primers used for qPCR are listed in Table S1.

**1.27 *In vivo* immune response**

In the wound model of MRSA infections, the wound tissues in different treatment groups were excised after 3 days of treatment. The collected soft tissues were placed in sterile precooling PBS and then digested and filtered through a 40 μm cell strainer. The resulting cell suspension was stained with antibodies against F4/80, CD86, and CD206 to analyze macrophage cell activity, according to the manufacturer’s instructions. Moreover, a high-speed tissue grinder was employed to homogenize the tissues via bead beating. The homogenates were centrifuged for 5 min, and the liquid supernatants were collected for further ELISA analysis. The concentrations of different cytokines were evaluated using the corresponding mouse Proteintech ELISA Kit (Proteintech Group, Inc., USA), according to the manufacturer’s instructions.

**1.28 *In vivo* CO detection in infected tissue**

The rat-infected model of MRSA infections was arranged with different treatments, as follows: (I) PBS+US; (II) SC gel; (III) S3fC gel; (IV) SC gel+US; (V) S3fC gel+US. After 1 h, the CO probe 1 dye (4.5 μg/g body weight) was injected into the infected sites and US 20 min. The fluorescence detection of CO in infected sites every 24 h was obtained using a IVIS Lumina XR III imaging system (PerkinElmer, Waltham, MA, USA).

Wound tissue samples were collected at indicated time points (0, 1, 2, 4, 8, 16, 24, 48, 96, 144, 192, 240 h) after treatment, immediately homogenized in ice‑cold PBS (100 mg tissue / 1 mL PBS), and centrifuged at 10,000 × g for 10 min at 4°C. The supernatant was used for CO detection. A commercial CO‑sensitive electrode (World Precision Instruments, Inc., ISO‑CO‑2) was calibrated using standard CO solutions prepared from a saturated CO stock solution (3.0 mM at 25°C). The electrode was connected to a picoammeter, and the current response was recorded. To ensure accuracy, a standard curve was also established by gas chromatography (Agilent 7890B, USA) using certified CO gas standards (1–50 ppm). The electrode readings were corrected against the GC‑derived standard curve. Each sample was measured in triplicate, and the CO concentration was expressed as micromolar (μM) based on the calibration curve.

**1.29 *In vivo* NETs expression**

After SC gel and S3fC gel treatment, rat skin tissues were homogenized in RIPA lysis buffer supplemented with protease and phosphatase inhibitors. After centrifugation, protein concentrations were determined using a BCA assay. Equal amounts of protein (20-30 µg) were separated by SDS-PAGE and transferred to PVDF membranes. Membranes were blocked and then incubated overnight at 4°C with primary antibodies against Cit-H3 (Cell Signaling 40503), MPO (Abcam ab208670), and PAD4 (Abcam ab214810, RRID:AB_3678629), followed by incubation with appropriate HRP-conjugated secondary antibodies. Protein bands were visualized using enhanced chemiluminescence, with GAPDH serving as a loading control.

**1.30 *In vivo* vessel detection**

The procedure for *in vivo* vessel detection began with the systemic administration of Alexa Fluor 647-conjugated anti-CD31 antibody (Abcam) to SD rats previously treated with either the CO depot or PBS, allowing 15-20 minutes of circulation for complete vascular labeling. Following perfusion, the target tissues were harvested and fixed in 4% paraformaldehyde. For 3D analysis, tissues were cleared using an established protocol and subsequently immunostained with a cyanine dye, FD-1080-conjugated anti-α-SMA antibody (Abcam ab124964, RRID:AB_11129103) to assess pericyte coverage. High-resolution Z-stack images of the prepared samples were acquired using a Nikon A1R confocal laser scanning microscope. The resulting image datasets were used to perform 3D reconstruction of the CD31-positive vasculature, and vascular maturity was quantified by calculating the percentage of the CD31^+^ vessel surface area that was co-localized with the α-SMA signal.

**1.31 *In Vivo* Pharmacokinetics and Biodistribution of Mn**

Male Sprague‑Dawley rats (~12 weeks old) were injected intravenously with 3HF@COFQDs (10 mg/kg). Blood samples were collected at 0, 5, 10, 24, 48, and 96 h post‑injection via retro‑orbital bleeding. At 0, 5, 10, 24, and 48 h, rats were euthanized, and organs (heart, liver, spleen, lung, kidney, and brain) were harvested. Urine and feces were collected at 0.5, 1, 5, 10, and 15 days. To assess long‑term residual Mn, organs, urine, and feces were also harvested from a separate group of rats at 15 days post‑injection. Samples (blood, organ homogenates, urine, feces) were digested in concentrated HNO_3_/H_2_O_2_ (3:1) at 120°C for 4 h, diluted with ultrapure water, and analyzed for Mn content by inductively coupled plasma mass spectrometry (ICP‑MS; Agilent 7800; Agilent Technologies, USA). Mn concentration in each sample was calculated from a standard curve and normalized to organ weight or volume. For pharmacokinetic analysis, blood Mn concentration‑time data were fitted using a non‑compartmental model DAS software (Version 2.0, RRID:SCR_022672).

**2. Figures of Results**

**Figure S1.** XANES and EXAFS spectra of COFQDs (Mn doped).

**Figure S2.** a) Nitrogen sorption isotherm and Pore size distribution (PSD) profiles of COFs. b) Nitrogen sorption isotherm and Pore size distribution (PSD) profiles of 3HF@COFQDs. COFs and 3HF@COFQDs activated at 120 °C for 24 h.

**Figure S3.** Digital photos of PBS and COFQDs solutions showing the Tyndall effect.

**Figure S4.** ζ (Zeta)-potential of COFs, COFQDs, and 3HF@COFQDs.

**Figure S5.** UV-Vis spectra of COFQDs in a weak acid aqueous solution at 0 days and 7 days.

**Figure S6.** FT-IR spectra of COFQDs and 3HF@COFQDs.

**Figure S7.** a-c) High-resolution XPS spectra in C 1s, N 1s, and O 1s regions of the COFQDs.

**Figure S8.** Ultrasonic current of 3HF@COFQDs under continuous US irradiation.

**Figure S9.** HRMS spectra of the 3HF@COFQDs eluent without and after US irradiation. [3HF] = 239.0689 m/z, [3HF+H] = 240.0729 m/z, [3HF-CO] = 243.2863 m/z, [3HF-CO+H] = 244.2947 m/z.

**Figure S10.** GS chromatograms of the CO standard gas and 3HF@COFQDs after US irradiation.

**Figure S11.** In situ DRIFTS spectra of 3HF@COFQDs under US irradiation during 120 min.

**Figure S12.** Live-dead staining images of E. coli and MRSA. Green represents SYTO9, and red represents PI.

**Figure S13.** Crystal violet staining images after treatment with PBS (Control), COFQDs, 3HF@COFQDs, COFQDs+US, and 3HF@COFQDs+US.

**Figure S14.** H&E staining images of native SIS and decellularized SIS. The blue represents the nucleus, and the red represents the ECM.

**Figure S15.** Contents of DNA, GAG, and collagen in native SIS (nSIS), SIS gel, S3fC gel. n = 5. Data are expressed as mean ± SD, * denotes *P* < 0.05, ** denotes *P* < 0.01, *** denotes *P* < 0.001, *P* values are calculated using two-sided *t*-test.

**Figure S16.** a) Representative digital photographs of S3fC gel degradation at 0, 120, and 240 h. b) Degradation rates of S3fC gel in a simulated physiological environment (0.25 mg/mL collagenase) at different time points. n = 3. Data are expressed as mean ± SD.

**Figure S17.** Inhibitory effects of SIS gel, SC gel, and S3fC gel against MRSA under different US power. n = 5. Data are expressed as a scatter plot (show all points), * denotes *P* < 0.05, ** denotes *P* < 0.01, *P* values are calculated using two-sided *t*-test.

**Figure S18.** a,b) The mean fluorescence intensity of CO and ROS, quantified from confocal images. n = 5. Data are expressed as mean ± SD, *** denotes *P* < 0.001, *P* values are calculated using two-sided *t*-test.

**Figure S19.** Cell viability of HUVECs measured by MTT for PBS (Control) and different concentrations of COFs, COFQDs, 3HF@COFQDs, as well as SIS gel, SC gel, and S3fC gel. n = 5. Data are expressed as mean ± SD.

**Figure S20.** Transwell images of PBS (Control), SC gel, S3fC gel, SC gel+US, and S3fC gel+US groups.

**Figure S21.** Cell proliferation imaging of PBS (Control), SC gel, S3fC gel, SC gel+US, and S3fC gel+US groups. Metabolism and proliferation by detecting intracellular esterase activity in live cells using Calcein AM probe.

**Figure S22.** Quantification of cell migration at 24 (left) and 48 (right) hours post-treatment in PBS (Control), SC gel, S3fC gel, SC gel+US, and S3fC gel+US groups. n = 3. Data are expressed as mean ± SD, *** denotes *P* < 0.001, *P* values are calculated using two-sided *t*-test.

**Figure S23.** Hemolysis ratio of PBS (Negative Control), Triton X100 (Positive Control), SIS gel, SC gel, and S3fC gel. n = 5. Data are expressed as mean ± SD.

**Figure S24.** Changes in DEGs of the Toll-like receptor sigaling pathways between the Control and S3fC gel+US groups.

**Figure S25.** The change of body weight for MRSA-infected acute mice after various treatments. n = 5. Data are expressed as mean ± SD. Notably, the body weight loss of all mice did not exceed 15% of their initial body weight throughout the entire study period.

**Figure S26.** Collagen volume fraction for the wound tissue in MRSA acute-infected mice with different treatments. n = 5. Data are expressed as mean ± SD, *** denotes *P* < 0.001, *P* values are calculated using two-sided *t*-test.

**Figure S27.** Inflammatory cell counts in wound tissues across different treatment groups via H&E staining.

**Figure S28.** Contour plot of chronic wound closure area for different groups at days 0, 1, 3, 5, 8, and 15.

**Figure S29.** The change of body weight for MRSA-infected chronic rats after various treatments. n = 5. Data are expressed as mean ± SD. Notably, the body weight loss of all rats did not exceed 15% of their initial body weight throughout the entire study period.

**Figure S30.** Flow cytometry analysis after co-incubation with CO probe in PBS (Control), SC gel, S3fC gel, SC gel+US, and S3fC gel+US.

**Figure S31.** Quantitative measurement of local CO concentration using a CO‑sensitive electrode. a) Schematic diagram of the experimental setup for detecting CO in wound tissue homogenates. b) Calibration curves: GC standard curve and the CO electrode calibration curve after correction. c) Time‑dependent CO concentrations in wound tissue of the S3fC gel+US group and the PBS control group over 240 h. n = 3. Data are presented as mean ± SD.

**Figure S32.** a,b) The mean fluorescence intensity for CD86^+^ and CD206^+^, quantified from immunofluorescent staining. n = 5. Data are expressed as mean ± SD, ** denotes *P* < 0.01, *** denotes *P* < 0.001, *P* values are calculated using two-sided *t*-test.

**Figure S33.** H&E staining images of chronic infected wounds collected from different treatment groups on day 15.

**Figure S34.** a) Blood COHb levels over 15 days after treatment. b) Serum HO-1 levels over 15 days. n = 5. Data are presented as mean ± SD.

**Figure S35.** a) Blood Mn concentration-time curve over 96 h post-injection. b) Mn levels in heart, liver, and kidney within 48 h. c) Mn levels in lung, spleen, and brain within 48 h. n = 5. Data are presented as mean ± SD.

**Figure S36.** a) Residual Mn concentrations in heart, liver, spleen, lung, and kidney at 15 days post-injection. n = 5. b) Mn levels in urine and feces at various time points over 15 days. n = 3. Data are presented as mean ± SD.

**Figure S37.** Representative H&E staining images of major organs (heart, liver, spleen, lung and kidney) collected from different treatment groups.

**Figure S38.** a-f) Hematology and clinical chemistry results for 0, 3, 10, and 15-day mice exposed to S3fC gel+US. n = 5. Data are presented as mean ± SD.

**Figure S39.** a-c) Serum biochemical indices at day 15 post‑injection. Levels of alanine aminotransferase (ALT), aspartate aminotransferase (AST), and blood urea nitrogen (BUN) in rats treated with S3fC gel+US compared with the healthy control group. d-f) Serum electrolyte levels at day 15 post‑injection. Concentrations of Ca^2+^, K^+^, and Cl^-^ in rats treated with S3fC gel+US versus the healthy control group. g-i) Blood gas parameters at day 15 post‑injection. Partial pressure of carbon dioxide (pCO_2_), partial pressure of oxygen (pO_2_), and pH in rats treated with S3fC gel+US compared with the healthy control group. n = 5. Data are presented as mean ± SD.

**Table S1.** Primers for qPCR assay in cell-based experiments.

| RNA | Sequences (from 5’ to 3’) |
| --- | --- |
| IL-6 | Forward primer: CTGCAAGAGACTTCCATCCAG |
|  | Reverse primer: AGTGGTATAGACAGGTCTGTTGG |
| IL-1β | Forward primer: GAAATGCCACCTTTTGACAGTG |
|  | Reverse primer: TGGATGCTCTCATCAGGACAG |
| CXCL15 | Forward primer: TCGAGACCATTTACTGCAACAG |
|  | Reverse primer: CATTGCCGGTGGAAATTCCTT |
| COX5B | Forward primer: GGAAGACCCTAATCTAGTCCCG |
|  | Reverse primer: GTTGGGGCATCGCTGACTC |
| ATP5A1 | Forward primer: TCTCCATGCCTCTAACACTCG |
|  | Reverse primer: CCAGGTCAACAGACGTGTCAG |
| SDHB | Forward primer: ATTTACCGATGGGACCCAGAC |
|  | Reverse primer: GTCCGCACTTATTCAGATCCAC |
| UQCRC2 | Forward primer: AAAGTTGCCCCGAAGGTTAAA |
|  | Reverse primer: GAGCATAGTTTTCCAGAGAAGCA |
| MAPK8 | Forward primer: GTGGAATCAAGCACCTTCACT |
|  | Reverse primer: TCCTCGCCAGTCCAAAATCAA |
| MAPK14 | Forward primer: TGACCCTTATGACCAGTCCTTT |
|  | Reverse primer: GTCAGGCTCTTCCACTCATCTAT |
| FOS | Forward primer: CGGGTTTCAACGCCGACTA |
|  | Reverse primer: TGGCACTAGAGACGGACAGAT |
| JUN | Forward primer: TTCCTCCAGTCCGAGAGCG |
|  | Reverse primer: TGAGAAGGTCCGAGTTCTTGG |

**References**

[1] Sun B, Ye Z, Zhang M, et al. Light-activated biodegradable covalent organic framework-integrated heterojunction for photodynamic, photothermal, and gaseous therapy of chronic wound infection. *ACS Appl. Mater. Interfaces*, 2021, 13(36): 42396-42410.

[2] Luo J C, Chen W, Chen X H, et al. A multi-step method for preparation of porcine small intestinal submucosa (SIS). *Biomaterials*, 2011, 32(3): 706-713.

[3] Gao L, Cheng J, Shen Z, et al. Orchestrating nitric oxide and carbon monoxide signaling molecules for synergistic treatment of MRSA infections. *Angew. Chem. Int. Ed.*, 2022, 61(3): e202112782.

[4] Kong J, Wang Y, Liu Y, et al. Antimicrobial and anti-inflammatory effects of antimicrobial peptide Lf-KR against carbapenem-resistant *Escherichia coli*. *BMC Microbiol.*, 2025, 25(1): 183.

[5] Su Y, Zhang X, Ren G, et al. In situ implantable three-dimensional extracellular matrix bioactive composite scaffold for postoperative skin cancer therapy. *Chem. Eng. J.*, 2020, 400: 125949.
